# Increased mortality in elderly patients with acute respiratory distress syndrome is not explained by host response

**DOI:** 10.1186/s40635-019-0270-1

**Published:** 2019-10-29

**Authors:** Laura R. A. Schouten, Lieuwe D. J. Bos, A. Serpa Neto, Lonneke A. van Vught, Maryse A. Wiewel, Arie J. Hoogendijk, Marc J. M. Bonten, Olaf L. Cremer, Janneke Horn, Tom van der Poll, Marcus J. Schultz, Roelie M. Wösten-van Asperen, the MARS consortium, F. M. de Beer, L. D. Bos, G. J. Glas, J. Horn, A. J. Hoogendijk, R. T. van Hooijdonk, M. A. Huson, T. van der Poll, B. P. Scicluna, L. R. A. Schouten, M. J. Schultz, M. Straat, L. A. van Vught, L. Wieske, M. A. Wiewel, E. Witteveen, M. J. M. Bonten, O. L. Cremer, J. F. Frencken, K. van de Groep, P. M. Klein Klouwenberg, M. E. Koster-Brouwer, D. S. Ong, D. M. Verboom

**Affiliations:** 1 Department of Pediatric Intensive Care, Academic Medical Center, University of Amsterdam, Amsterdam, the Netherlands; 2 Department of Intensive Care, Academic Medical Center, University of Amsterdam, Amsterdam, The Netherlands; 3 Laboratory of Experimental Intensive Care and Anesthesiology (L·E·I·C·A), Academic Medical Center, University of Amsterdam, Amsterdam, the Netherlands; 4 Department of Critical Care Medicine, Hospital Israelita Albert Einstein, São Paulo, Brazil; 5 Center of Experimental and Molecular Medicine (CEMM), Academic Medical Center, University of Amsterdam, Amsterdam, the Netherlands; 6 Department of Medical Microbiology, University Medical Center Utrecht, Utrecht, the Netherlands; 7 Department of Intensive Care, University Medical Center Utrecht, Utrecht, the Netherlands; 8 Mahidol–Oxford Tropical Medicine Research Unit (MORU), Mahidol University, Bangkok, Thailand; 9 Department of Pediatric Intensive Care, University Medical Center Utrecht, Utrecht, the Netherlands

**Keywords:** Critical care, intensive care, ARDS, host response, Aging, Outcome, Biomarker, Mediation

## Abstract

**Background:**

Advanced age is associated with increased mortality in acute respiratory distress syndrome (ARDS) patients. Preclinical studies suggest that the host response to an injurious challenge is age-dependent. In ARDS patients, we investigated whether the association between age and mortality is mediated through age-related differences in the host response.

**Methods:**

This was a prospective longitudinal observational cohort study, performed in the ICUs of two university-affiliated hospitals. The systemic host response was characterized in three predefined age-groups, based on the age-tertiles of the studied population: young (18 to 54 years, *N* = 209), middle-aged (55 to 67 years, *N* = 213), and elderly (67 years and older, *N* = 196). Biomarkers of inflammation, endothelial activation, and coagulation were determined in plasma obtained at the onset of ARDS. The primary outcome was 90-day mortality. A mediation analysis was performed to examine whether age-related differences in biomarker levels serve as potential causal pathways mediating the association between age and mortality.

**Results:**

Ninety-day mortality rates were 30% (63/209) in young, 37% (78/213) in middle-aged, and 43% (84/196) in elderly patients. Middle-aged and elderly patients had a higher risk of death compared to young patients (adjusted odds ratio, 1.5 [95% confidence interval 1.0 to 2.3] and 2.1 [1.4 to 3.4], respectively). Relative to young patients, the elderly had significantly lower systemic levels of biomarkers of inflammation and endothelial activation. Tissue plasminogen activator, a marker of coagulation, was the only biomarker that showed partial mediation (proportion of mediation, 10 [1 to 28] %).

**Conclusion:**

Little evidence was found that the association between age and mortality in ARDS patients is mediated through age-dependent differences in host response pathways. Only tissue plasminogen activator was identified as a possible mediator of interest.

**Trial registration:**

This trial was registered at ClinicalTrials.gov (identifier NCT01905033, date of registration July 23, 2013).

## Introduction

Epidemiological data and preclinical studies using animal models of lung injury show that advanced age is associated with increased susceptibility to develop the acute respiratory distress syndrome (ARDS) [1–3]. Age has also been recognized as one of the major determinants predicting morbidity and mortality in patients with ARDS [4, 5]. Even though elderly patients usually have more comorbidities, this does only partially explain their higher burden of disease [1]. In view of the growing number of elderly patients in intensive care units (ICUs) [6], recognition as well as understanding of the association between advanced age and adverse outcome in ARDS patients could improve prognostication and may even allow for development of age-specific treatment strategies.

Responses to tissue injury in general are modified by the process of aging [7, 8]. In healthy humans, age-related changes in the immune system, including an increased release of pro-inflammatory cytokines and chemokines [7–9], coagulation factors [10] and acute phase reactants, dysregulation of the activation and migration of inflammatory cells [11, 12], and endothelial dysfunction [13] have all been associated with functional decline and increased mortality. In addition, there is some evidence that this chronic activation of the “aged immune system” results in an uncontrolled host response to injury, an impairment to mount pathogens, and an inability to resolve tissue damage [8]. Little is known about the impact of this sometimes called “inflamm-aging” or “immunosenesence” on development and progression of ARDS [14]. Preclinical studies have shown a progressive pro-inflammatory status and an altered response to direct and indirect pulmonary insults in elderly animals [3, 15, 16], yet no studies in ARDS patients have been performed to confirm this in the human setting. Those clinical studies that did compare levels of inflammation and coagulation between age groups focused on specific subgroups of critically ill patients and showed conflicting results [17–23]. In particular, none of these studies investigated age-related differences in the host response as a potential mediator of the outcome. Studying the biological host response using a statistical mediation analysis can provide an etiological understanding of the association between age and outcomes in ARDS patients [24].

This study aimed to characterize and compared the systemic host response in ARDS patients in three age groups and investigated whether the association between age and mortality in ARDS patients is mediated through age-dependent differences in host responses. It was hypothesized that an aggravated host response, i.e., a host response with an excessive increase of one or more systemic biomarkers known to be involved in inflammation, endothelial activation, or coagulation, at least partially explains the increased mortality in elderly with ARDS.

## Methods

### Study design and setting

This study was performed as a preplanned secondary analysis of the “Molecular Diagnosis and Risk stratification of Sepsis” (MARS) Biobank project, a prospective observational cohort study performed in the mixed ICUs of two university-affiliated hospitals in the Netherlands (ClinicalTrials.gov identifier NCT01905033) [25–29]. The Institutional Review Board of both study centers approved the study protocol (protocol no. 10–056C) with an opt-out informed consent method. Part of the patients’ cohort and results of biomarkers’ measurements have been used in previous reports on the MARS study [25–34].

### Inclusion and exclusion criteria

The parent MARS study included consecutive patients admitted to the participating ICUs if expected to stay in the unit beyond the following calendar day. The current study restricted participation to patients having acute lung injury (ALI) or ARDS, according to the American-European Consensus Conference (AECC) criteria [35] as used at the time the MARS study ran. Trained researchers collected data prospectively and screened all patients daily for the presence of ALI/ARDS. After closing the MARS study, patients were re-classified as having mild, moderate, or severe ARDS, according to the Berlin definition [36], based on the PaO_2_ to FiO_2_ ratio at the day of ARDS diagnosis. Patients with a limitation on life-sustaining measures at ICU admission were excluded, patients in whom biomarkers were not determined, as were patients aged under 18 years.

### Age groups

The cohort of patients was divided into three groups using cut-off levels based on age-tertiles in the total cohort: young adults (18 to 54 years), middle-aged adults (55 to 67 years), and elderly (> 67 years and older).

### Endpoints

The primary endpoint was 90-day mortality, defined as death within 90 days after the onset of ARDS. Secondary outcome measures were 30-day mortality, 1-year mortality, ICU mortality, in-hospital mortality, ventilator-free days and alive at day 28 (VFD), ICU length of stay (ICU-LOS), ICU-free days and alive at day 30 (ICU-FD), in-hospital length of stay (hosp-LOS) and hospital-free days, and alive at day 90 (hosp-FD). The occurrence of death was recorded prospectively if patients died at the ICU. In addition, for all patients, we assessed the vital status in the government registration of persons at 1 year after admission to the ICU. In case a patient was deceased, the specific date of death was recorded. Other patient data was collected prospectively, for details on data collection and definitions, see Additional file 1.

### Biomarker measurements

Daily left-over EDTA anti-coagulated plasma was harvested from blood obtained for regular patient care [28, 29]. For the current analysis, the sample nearest to the day of ARDS diagnosis was used. A panel of 20 biomarkers known to be involved in inflammation, endothelial activation, and coagulation pathways (e.g., interleukin (IL)-6, IL-8, IL-10, IL-1β, tumor necrosis factor-alpha (TNF-α), interferon-gamma (INF-γ), intracellular adhesion molecule (ICAM)-1, matrix metalloproteinase (MMP)-8, metallopeptidase inhibitor (TIMP)-1, fractalkine, E-selectin, P-selectin, angiopoietin-1 (ANG1) and ANG2, platelet factor 4 (PF4), protein C, plasminogen activator inhibitor (PAI)-1, antithrombin (AT), d-dimer, and tissue plasminogen activator (tPA) were measured [37–39]. For assays, see Additional file 1 and Additional file 1: Table S1.

### Statistical analysis

First, clinical characteristics and outcomes were compared for the three age groups. Data are presented as absolute numbers with proportions, medians with interquartile ranges, or means with standard deviations, as appropriate. Differences between the age groups were analyzed using Kruskal-Wallis test for continuous variables and chi-squared for categorical variables. Survival differences between age groups were visualized by Kaplan-Meier plots and tested with a log-rank test.

Next, the association between age and mortality was analyzed by univariate and multiple logistic regression, the confounders included are described later in the “Methods” section. Then, the host response was compared for the levels of the 20 biomarkers between young adults and middle-aged adults, and young adults and elderly using Mann-Whitney *U* tests.

**Fig. 1:**
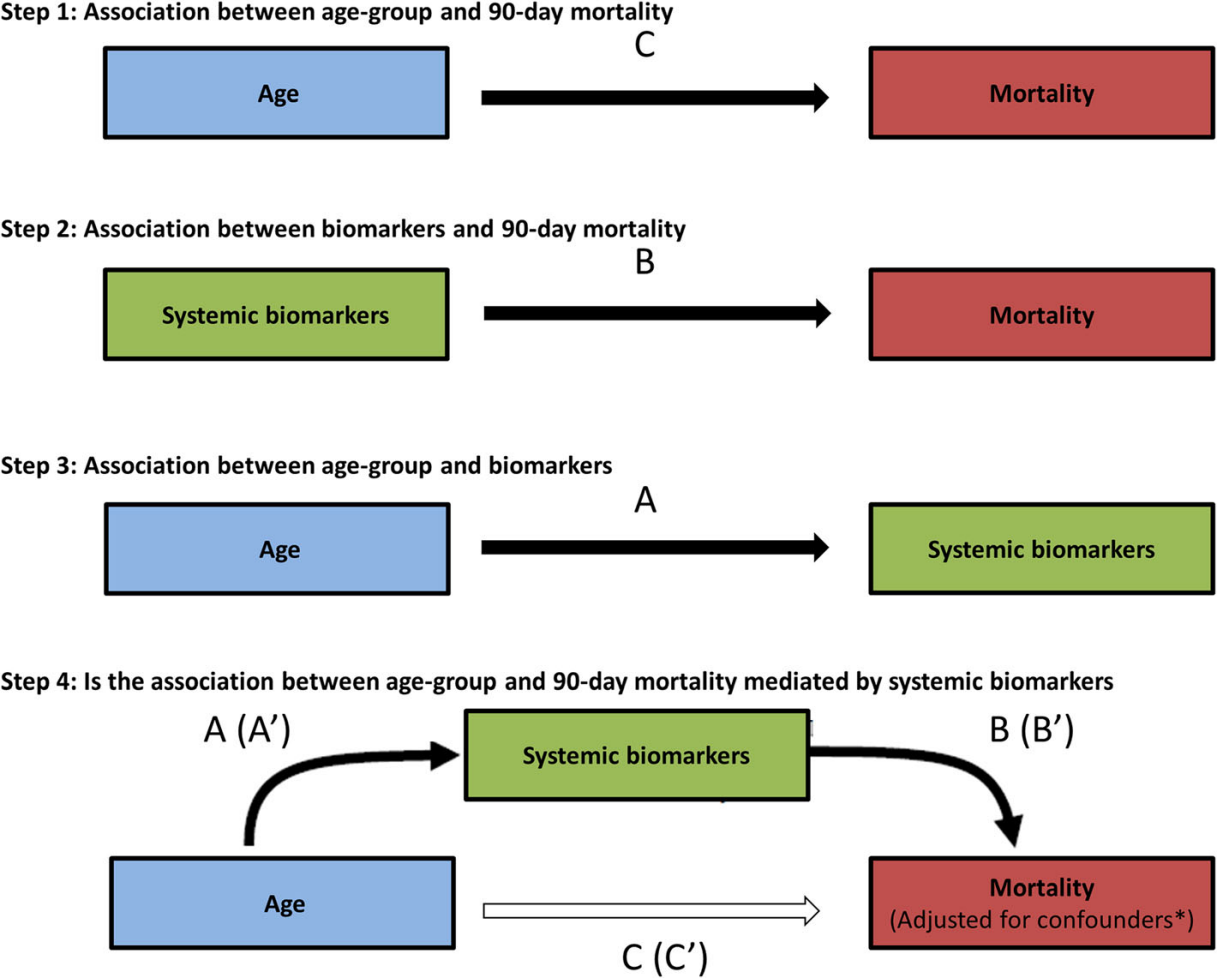
Pathway analysis: stepwise mediation analysis assessing whether the association between age and mortality is mediated by age-dependent differences in biomarker levels. *Adjusted for ethnic background, gender, admission type, readmission, direct hit for ARDS, Charlson Comorbidity Index, APACHE-IV score adjusted for age, immunodeficiency, tidal volume per predicted body weight, positive end-expiratory pressure. C = average direct effect (ADE); A*B = average causal mediation effect (ACME). A’, B’, and C’—adjusted for confounders

Finally, a mediation analysis was performed to investigate whether the association between the age groups and 90-day mortality was mediated through age-related differences in systemic biomarker levels (mediation step 1, Fig. 1). The mediation analysis is explained in more detail in Additional file 1. Briefly, first, the association between biomarker levels and 90-day mortality was tested using logistic regression models per each biomarker (mediation step 2). Then, the associations between the age-group and biomarker levels were determined with linear models (mediation step 3). In case of a significant association between age and a biomarker, and between that biomarker and mortality, the average direct effect (ADE) of age on a 90-day mortality and the effect of age on a 90-day mortality mediated by the biomarker were modeled (average causal mediation effect, ACME) (mediation step 4). Mediation was expressed as the ratio ACME to the total effect, so-called proportion of mediation. The 95% confidence intervals of the effects were obtained via bootstrapping. Mediation was only considered relevant if the point estimate of the mediated effect was in the same direction as the total effect because “negative mediation” can per definition not explain the increased mortality [40]. A priori < 5% was considered as a small proportion of mediation, 5–20% as a moderate and > 20% as large proportions of mediation [40, 41]. Young adults were defined as the reference group. Biomarkers were log-transformed to obtain normally distributed variables, and no outlines were removed from the analysis. All associations were determined with both univariate and multiple regression models, and effects were expressed as odds ratios (OR) or β-coefficients with 95% confidence intervals [95% CI], as appropriate. For multiple linear regression models, we reported the adjusted *R*^2^. In multiple logistic regression models, the goodness of fit was tested with the omnibus test. A *p* value < 0.05 was considered lack of fit.

Because an etiological mediation model was built, it was important to adjust for confounders of the exposure–outcome relation (age and mortality) and the mediator–outcome relation (biomarker levels and mortality) [40]. Potential confounders were selected according to biological plausibility, including ethnic background, gender, admission type, readmission, direct-hit for ARDS, Charlson Comorbidity Index (CCI), Acute Physiology and Chronic Health Evaluation (APACHE)-IV score adjusted for age, immunodeficiency, tidal volume per predicted body weight, and positive end-expiratory pressure. These confounders were included in all models [42]. Collinearity was tested using the variance inflation factor. A value > 2 was considered to be collinear. If so, one of the covariates was restricted from the analyses. The handling of missing data is outlined in Additional file 1: Table S2. Of note, in a large proportion of patients the plasma concentrations of the cytokines TNF-α (64%), IL-1β (39%), and INF-γ (46%) were under the detection limit and were imputed with the LOD value which may introduce over- or underestimation of the estimates (see online Additional file 1: Table S1).

Several sensitivity analyses were performed. To potentially reduce the heterogeneity, the mediation analysis was repeated with a subgroup of patients with a direct hit for ARDS (i.e., pulmonary ARDS). In addition, because it can be argued that CCI and APACHE-VI are mediators instead of confounders, the adjusted mediation analysis was repeated without these variables as confounders. Furthermore, the mediation analysis was repeated with age as a continuous variable—for this analysis also, the goodness of fit was reported. Finally, the biomarker levels from a subset of patients with a sample collected at a later timepoint during ICU admission (4 to 10 days after the onset of ARDS) were explored, by comparing the median biomarker levels between the age-groups using Mann-Whitney *U* tests. Of note, the median time to sampling from the onset of ARDS did not differ according to age (see Additional file 1: Table S22).

No statistical power analysis was conducted prior to the study. The sample size was based on the available number of patients. All statistical tests were two-tailed and were performed in *R* statistics using the *R*-studio interface (www.r–project.org). The mediation analysis was performed using the “mediation package” [43]. In addition, a Benjamini-Hochberg correction for multiple comparisons was performed. A *p* value < 0.05 was considered as statistically significant.

## Results

**Fig. 2:**
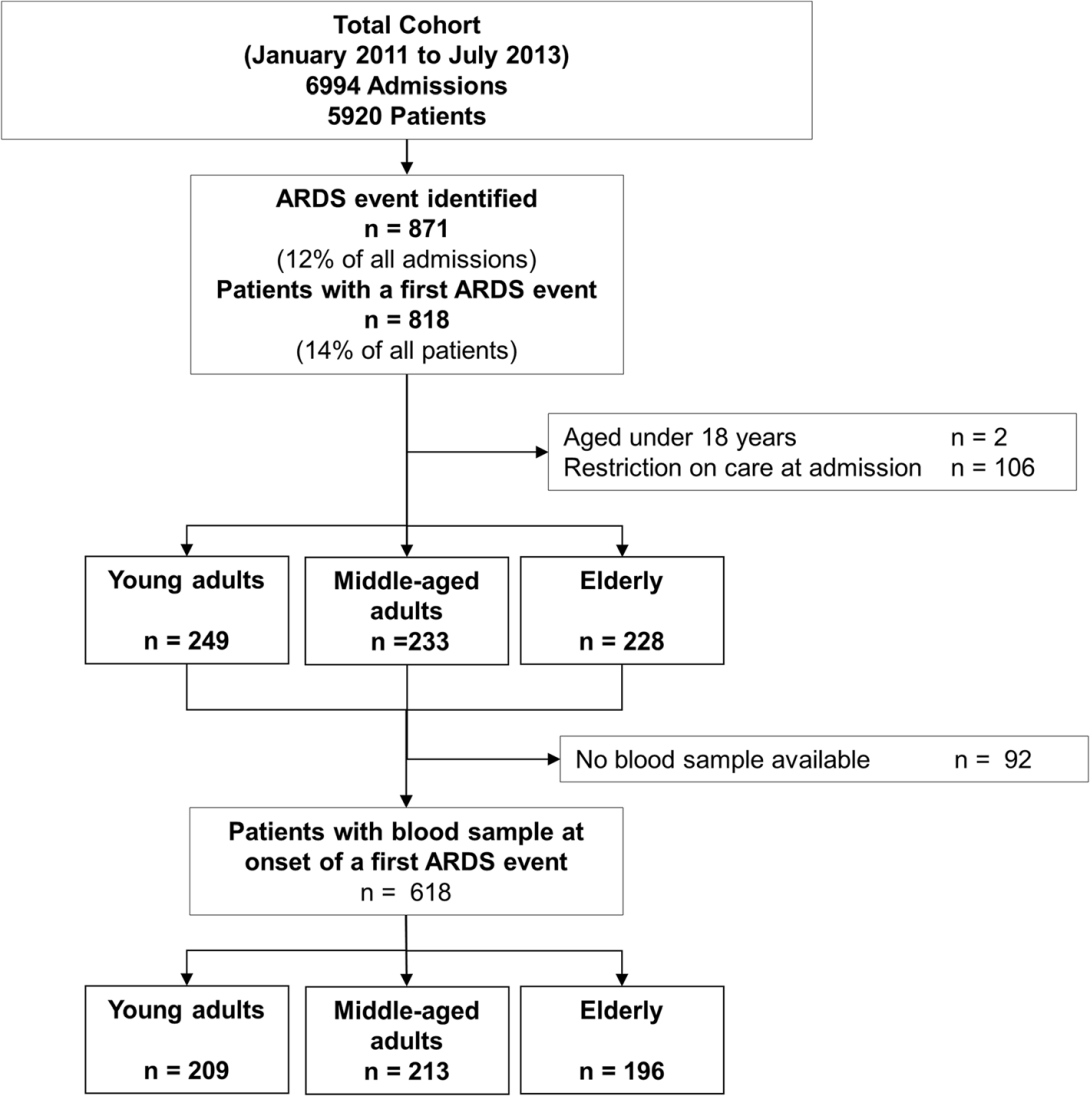
Patient flow chart

Of 818 ARDS patients included in MARS, two patients with an age under 18 years, 106 patients with a limitation on life-sustaining measures at ICU admission, and 92 patients without a blood sample were excluded, leaving 618 subjects for the full analysis (Fig. 2). Table 1 shows patient characteristics of 209 young, 213 middle-aged, and 196 elderly ARDS patients. In young patients, the prevalence of comorbidities was lower (see online Additional file 1: Table S3), but the severity of these chronic diseases among these patients was higher as reflected by a similar CCI among the age groups. In addition, young patients more frequently had an immune deficiency (*see* Additional file 1: Table S3).

**Table 1:** Characteristics of the ARDS patients

	Young adults (*n* = 209)		Middle-aged adults (*n* = 213)		Elderly (*n* = 196)		*p* value
Demographics							
Age (years), Median [IQR]	44	[36, 50]	61	[58, 64]	74	[71, 77]	NA
Male, *n*/total *n* (%)	119/209	(57)	135/213	(63)	134/196	(68)	0.060
Race, Caucasian, *n*/total *n* (%)	165/201	(82)	190/208	(91)	188/191	(98)	0.000
Admission							
Admission type, medical, *n*/total *n* (%)	160/209	(77)	142/213	(67)	129/196	(66)	0.037
Readmission, *n*/total *n* (%)	22/209	(11)	27/213	(13)	31/196	(16)	0.285
Charlson Comorbidity Index, Median [IQR]	0	[0, 2]	0	[0, 2]	0	[0, 2]	0.288
APACHE-IV score, median [IQR]	76	[58, 101]	80	[61, 104]	85	[67, 102]	0.014
APACHE-IV score adjusted for age, median [IQR]	73	[56, 100]	67	[50, 90]	68	[50, 85]	0.150
Lung injury prediction score, median [IQR]	9	[7, 10]	9	[7, 10]	8	[7, 10]	0.472
ARDS diagnosis							
Direct hit for ARDS^*^, *n*/total *n* (%)	142/208	(68)	137/211	(65)	125/192	(65)	0.619
Berlin classification							
Mild, *n*/total *n* (%)	84/209	(40)	78/213	(37)	84/196	(43)	0.409
Moderate, *n*/total *n* (%)	98/209	(47)	97/213	(46)	87/196	(44)	0.876
Severe, *n*/total *n* (%)	27/209	(13)	38/213	(18)	25/196	(13)	0.248
ARDS on admission, *n*/total *n* (%)	165/209	(79)	153/213	(72)	157/196	(80)	0.089
Time to ARDS from ICU admission, median [IQR]	0	[0, 1]	0	[0, 2]	0	[0, 1]	0.163
Organ failure							
SOFA score at onset of ARDS, median [IQR]	8	[5, 11]	8	[6, 11]	8	[6, 10]	0.768
Acute kidney injury during admission, *n*/total *n* (%)	106/209	(51)	122/213	(57)	123/196	(63)	0.054
Ventilation and oxygenation at onset of ARDS							
Tidal volume per PBW, median [IQR]	7.1	[6.2, 8.2]	7.2	[6.3–9.0]	7.1	[6.3, 8.1]	0.357
PEEP (cmH_2_O), median [IQR]	10	[8, 14]	10	[8, 12]	8	[6, 12]	0.025
Inspiratory peak pressure (cmH_2_O), median [IQR]	27	[20, 33]	25	[19, 31]	24	[19, 30]	0.036
PaO_2_ FiO_2_ ratio, median [IQR]	171	[121, 219]	160	[106, 216]	164	[120, 223]	0.432

*SOFA* sequential organ failure assessment, *IQR* interquartile range, *APACHE* Acute Physiology and Chronic Health Evaluation, *PBW* predicted body weight, *PEEP* positive end-expiratory pressure, *NA* not applicable. A *p* value < 0.05 was considered as statistically significant. ^*^Supplementary data provides a table with detailed data on predisposing factors (see online Additional file 1: Table S1)

The 90-day mortality rate was 36% (225/618) for the entire cohort, 30% (63/209) in young patients, 37% (78/213) in middle-aged patients, and 43% (84/196) in elderly patients. Middle-aged and elderly patients had 1.5 to 2.1 times higher risk of dying within 90 days after the onset of ARDS compared to young patients, after adjustment for confounders (Table 2, see online Additional file 1: Figure S1). Similar age-related differences existed for 1-year mortality, in-hospital mortality, and hospFD (Table 2, Additional file 1: Figure S1, Table S4, Table S5). There were no significant differences in ICU mortality, 30-day mortality, VFD, ICU-LOS, ICU-FD, and hosp-LOS (see Additional file 1: Table S4, Table S5). Of note, the SOFA scores and the limitation on life-sustaining measures at the time of death of the patients that died in the ICU did not differ according to age (see Additional file 1: Table S6).

**Table 2:** Association between age and mortality in ARDS patients *(mediation step 1)*

	Crude odds ratio [95% CI]	*p* value	Adjusted odds ratio [95% CI]^‡^	*p* value	GOF *p* value
90-day mortality					
Young adults	Reference	NA	Reference	NA	0.153
Middle-aged adults	1.3 [0.9, 2.0]	0.159	1.5 [1.0, 2.3]	0.078	
Elderly	1.7 [1.2, 2.6]	0.008	2.1 [1.4, 3.4]	0.001	
30-day mortality					
Young adults	Reference	NA	Reference	NA	0.081
Middle-aged adults	1.1 [0.7, 1.7]	0.740	1.2 [0.8, 1.9]	0.469	
Elderly	1.3 [0.8, 2.0]	0.240	1.5 [0.9, 2.4]	0.096	
1-year mortality					
Young adults	Reference	NA	Reference	NA	0.716
Middle-aged adults	1.5 [1.0, 2.2]	0.035	1.7 [1.1, 2.6]	0.016	
Elderly	2.0 [1.4, 3.0]	0.000	2.4 [1.6, 3.8]	0.000	

Data is presented as odds ratio’s (OR) with a 95% confidence interval [CI]. ^‡^Adjusted for ethnic background, gender, admission type, readmission, direct hit for ARDS, Charlson Comorbidity Index, APACHE-IV score adjusted for age, immunodeficiency, tidal volume per predicted body weight, and positive end-expiratory pressure. A *p* value < 0.05 was considered as statistically significant. *GOF* goodness of fit, omnibus test, a *p* value of < 0.05 is considered as a lack of fit. *NA* not applicable

**Fig. 3:**
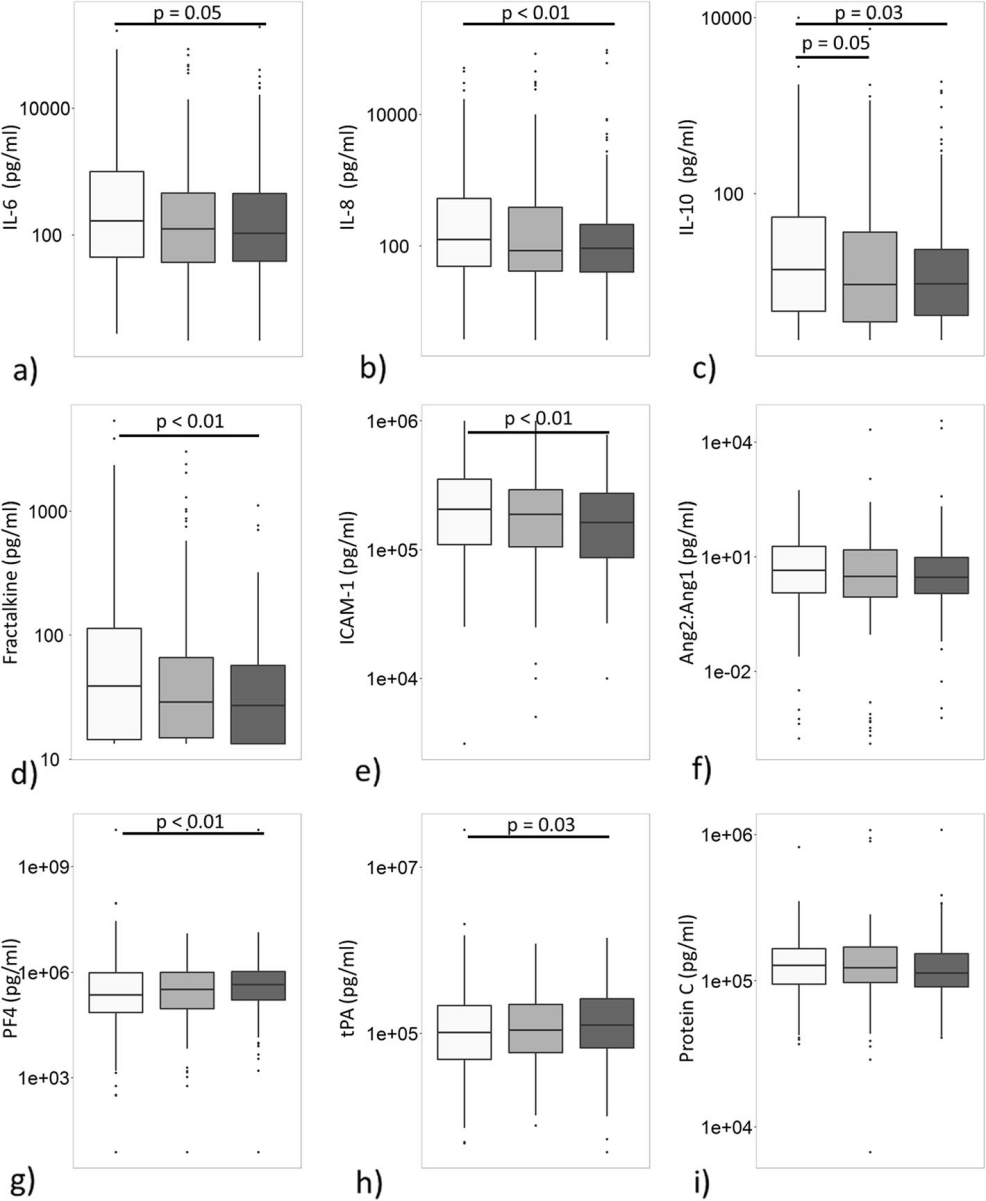
Systemic levels of biomarker in ARDS patients stratified by age group. Systemic levels of **a** interleukin (IL)-6, **b** IL-8, **c** IL-10, **d** Fractalkine, **e** intracellular adhesion molecule (ICAM)-1, **f** angiopoetin 2:angiopoetin 1 (ANG2:ANG1), **g** platelet factor (PF) 4, **h** tissue plasminogen activator (tPA), **i** protein C, at onset of ARDS.  Young adults,  middle-aged adults, and  elderly. Box and whisker diagrams depict the median and lower quartile, upper quartile, and their respective 1.5 IQR as whiskers—as specified by Tukey. Group differences between young adults and middle-aged adults, and young adults and elderly were tested by a Mann-Whitney *U* test. A *p* value < 0.05 was considered as statistical significant

Except for lower plasma levels of E-selectin and IL-10, no statistically significant differences in biomarker levels in plasma were found between young and middle-aged patients (Fig. 3). However, compared to young patients, elderly patients had lower plasma levels of IL-6, IL-8, IL-10, INF-γ, fractalkine, ICAM-1, E-selectin, and higher plasma levels of PF4 and tPA (Fig. 3, see Additional file 1: Figure S2).

Increased plasma levels of IL-8, IL-10, fractalkine, ANG2:ANG1, tPA, and PAI-1 were independently associated with 90-day mortality (see Additional file 1: Table S7). After adjusting for potential confounders, age was only statistically significant associated with increased levels of tPA and decreased levels of fractalkine and E-selectin (Tables 3, 4, and 5).

**Table 3:** Association between age and inflammatory marker levels in ARDS patients *(mediation step 3)*

	Coefficient [95% CI]	*p* value	Coefficient [95% CI]^*^	*p* value	Adjusted *R*^2^
IL-6					
Young adults	Reference	NA	Reference	NA	0.152
Middle-aged adults	− 0.33 [− 0.73, 0.08]	0.113	− 0.30 [− 0.69, 0.06]	0.117	
Elderly	− 0.40 [− 0.81, 0.01]	0.057	− 0.33 [− 0.73, 0.06]	0.100	
IL-8					
Young adults	Reference	NA	Reference	NA	0.247
Middle-aged adults	− 019 [− 0.54, 0.16]	0.478	− 0.06 [− 0.37, 0.26]	0.767	
Elderly	− 0.48 [− 0.84, − 0.12]	0.019	− 0.25 [− 0.58, 0.07]	0.148	
IL-10					
Young adults	Reference	NA	Reference	NA	0.203
Middle-aged adults	− 0.30 [− 0.62, 1.02]	0.174	− 0.21 [− 0.51, 0.08]	0.133	
Elderly	− 0.42 [− 0.75, − 0.09]	0.032	− 0.26 [− 0.57, 0.04]	0.058	
IL-1β					
Young adults	Reference	NA	Reference	NA	0.053
Middle-aged adults	− 0.06 [− 0.20, 0.09]	0.461	− 0.01 [− 0.16, 0.14]	0.854	
Elderly	− 0.00 [− 0.15, 0.15]	0.957	0.07 [− 0.09, 0.22]	0.392	
TNF-α					
Young adults	Reference	NA	Reference	NA	0.009
Middle-aged adults	− 0.04 [− 0.17, 0.08]	0.522	− 0.02 [− 0.15, 0.11]	0.671	
Elderly	0.05 [− 0.08, 0.18]	0.434	0.08 [− 0.06, 0.22]	0.248	
INF-γ					
Young adults	Reference	NA	Reference	NA	0.031
Middle-aged adults	− 0.16 [− 0.45, 0.12]	0.262	− 0.11 [− 0.39, 0.18]	0.524	
Elderly	− 0.35 [− 0.65, − 0.06]	0.017	− 0.24 [− 0.55, 0.06]	0.127	
MMP-8					
Young adults	Reference	NA	Reference	NA	0.044
Middle-aged adults	− 0.23 [− 0.63, 0.18]	0.271	− 0.19 [− 0.60, 0.21]	0.356	
Elderly	− 0.03 [− 0.45, 0.38]	0.869	− 0.01 [− 0.40, 0.41]	0.959	
TIMP-1					
Young adults	Reference	NA	Reference	NA	0.111
Middle-aged adults	− 0.08 [− 0.34, 0.18]	0.537	− 0.03 [− 0.29, 0.21]	0.776	
Elderly	− 0.13 [− 0.40, 0.13]	0.328	0.00 [− 0.26, 0.26]	0.996	

Data is presented as beta-coefficient (*β*) with a 95% confidence interval [CI]. All biomarkers are log-transformed. ^*^Adjusted for ethnic background, gender, admission type, readmission, direct hit for ARDS, Charlson Comorbidity Index, APACHE-IV score adjusted for age, immunodeficiency, tidal volume per predicted body weight, and positive end-expiratory pressure. A *p* value < 0.05 was considered as statistically significant. *IL* interleukin, *TNF-α* tumor necrosis factor alpha, *INF-γ*, interferon gamma, *MMP-8* matrix metalloproteinase-8, and *TIMP-1* metallopeptidase inhibitor-1. Goodness of fit, adjusted *R*^2^. *NA* not applicable

**Table 4:** Association between age and endothelial activation marker levels in ARDS patients *(mediation step 3)*

	Coefficient [95% CI]	*p* value	Coefficient [95% CI]^*^	*p* value	Adjusted *R*^2^
Fractalkine					
Young adults	Reference	NA	Reference	NA	0.193
Middle-aged adults	− 0.26 [− 0.49, − 0.04]	0.021	− 0.17 [− 0.38, 0.03]	0.103	
Elderly	− 0.46 [− 0.69, − 0.23]	< 0.001	− 0.26 [− 0.48, − 0.05]	0.018	
E-selectin					
Young adults	Reference	NA	Reference	NA	0.048
Middle-aged adults	− 0.31 [− 0.53, − 0.10]	0.005	− 0.27 [− 0.48, − 0.04]	0.013	
Elderly	− 0.24 [− 0.46, − 0.02]	0.032	− 0.21 [− 0.44, 0.02]	0.064	
P-selectin					
Young adults	Reference	NA	Reference	NA	0.047
Middle-aged adults	− 0.14 [− 0.36, 0.08]	0.214	− 0.09 [− 0.30, 0.13]	0.510	
Elderly	0.03 [− 0.20, 0.25]	0.818	0.05 [− 0.18, 0.27]	0.661	
ICAM-1					
Young adults	Reference	NA	Reference	NA	0.073
Middle-aged adults	− 0.11 [− 0.26, 0.04]	0.168	− 0.05 [− 0.20, 0.10]	0.547	
Elderly	− 0.22 [− 0.37, − 0.07]	0.005	− 0.14 [− 0.29, 0.02]	0.073	
Ang2:Ang1					
Young adults	Reference	NA	Reference	NA	0.046
Middle-aged adults	− 0.28 [− 0.73, 0.18]	0.232	− 0.29 [− 0.75, 0.15]	0.208	
Elderly	− 0.22 [− 0.68, 0.25]	0.359	− 0.19 [− 0.67, 0.28]	0.441	

Data is presented as beta-coefficient (*β*) with a 95% confidence interval [CI]. All biomarkers are log-transformed. ^*^Adjusted for ethnic background, gender, admission type, readmission, direct hit for ARDS, Charlson Comorbidity Index, APACHE-IV score adjusted for age, immunodeficiency, tidal volume per predicted body weight, and positive end-expiratory pressure. A *p* value < 0.05 was considered as statistically significant. *ICAM-1* intracellular adhesion molecule-1, *ANG-2:ANG1* angiopoetin-2:angiopoetin-1. Goodness of fit, adjusted *R*^2^. *NA* not applicable

**Table 5:** Association between age and coagulation marker levels in ARDS patients *(mediation step 3)*

	Coefficient [95% CI]	*p* value	Coefficient [95% CI]^*^	*p* value	Adjusted *R*^2^
Platelet factor 4					
Young adults	Reference	NA	Reference	NA	0.138
Middle-aged adults	0.26 [− 0.24, 0.76]	0.313	0.26 [− 0.21, 0.74]	0.262	
Elderly	0.59 [0.05, 1.08]	0.033	0.36 [− 0.14, 0.87]	0.129	
d-Dimer					
Young adults	Reference	NA	Reference	NA	0.031
Middle-aged adults	− 0.02 [− 0.22, 0.19]	0.857	− 0.01 [− 0.22, 0.20]	0.942	
Elderly	0.02 [− 0.19, 0.23]	0.829	0.03 [− 0.19, 0.24]	0.870	
Tissue plasminogen activator					
Young adults	Reference	NA	Reference	NA	0.063
Middle-aged adults	0.05 [− 0.15, 0.26]	0.520	0.08 [− 0.13, 0.28]	0.459	
Elderly	0.18 [− 0.04, 1.39]	0.103	0.24 [0.02, 0.45]	0.032	
PAI-1					
Young adults	Reference	NA	Reference	NA	0.141
Middle-aged adults	− 0.31 [− 0.64, 0.03]	0.072	− 0.21 [− 0.54, 0.10]	0.141	
Elderly	− 0.33 [− 0.68, 0.01]	0.054	− 0.21 [− 0.54, 0.12]	0.177	
Protein C					
Young adults	Reference	NA	Reference	NA	0.035
Middle-aged adults	− 0.00 [− 0.09, 0.09]	0.989	0.00 [− 0.09, 0.09]	0.973	
Elderly	− 0.05 [− 0.15, 0.04]	0.265	− 0.07 [− 0.17, 0.03]	0.204	
Anti-thrombin					
Young adults	Reference	NA	Reference	NA	0.066
Middle-aged adults	− 0.00 [− 0.13, 0.12]	0.992	0.01 [− 0.12, 0.13]	0.850	
Elderly	− 0.04 [− 0.82, 0.09]	0.564	− 0.03 [− 0.16, 0.10]	0.295	

Data is presented as beta-coefficient (*β*) with a 95% confidence interval [CI]. All biomarkers are log-transformed. ^*^Adjusted for ethnic background, gender, admission type, readmission, direct hit for ARDS, Charlson Comorbidity Index, APACHE-IV score adjusted for age, immunodeficiency, tidal volume per predicted body weight, positive end-expiratory pressure. A *p* value < 0.05 was considered as statistically significant. PAI-1, plasminogen activator inhibitor-1. Goodness of fit, adjusted *R*^2^. *NA* not applicable

**Fig. 4:**
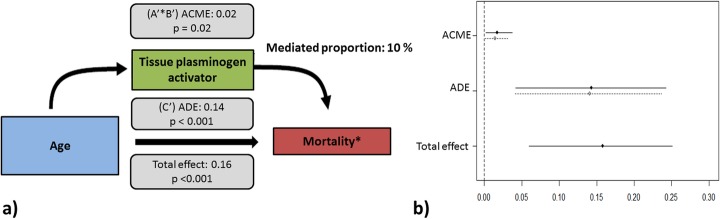
Mediation analysis. **a**, **b** Tissue plasminogen activator is a mediator which partially explains the association between age and mortality. *Adjusted for ethnic background, gender, admission type, readmission, direct hit for ARDS, Charlson Comorbidity Index, APACHE-IV score adjusted for age, immunodeficiency, tidal volume per predicted body weight, and positive end-expiratory pressure. ADE, average direct effect; ACME, average causal mediation effect. A *p* value < 0.05 was considered as statistical significant

Because age was significantly associated with IL-8, IL-10, fractalkine, PF4, PAI-1, and tPA (mediation step 2), and these biomarkers were significantly associated mortality (mediation step 3), either univariate or adjusted, this subset of biomarkers was tested as potential mediators attributing to the association between age and mortality (see Additional file 1: Table S8, Additional file 1: Table S9). Only tPA was found to be a significant mediator, mediating 10 [1 to 28] % (*p* = 0.018) of the association between age and mortality (Fig. 4). This means that the increased risk of death in the elderly 90 days after the onset of ARDS is partially explained by their higher systemic levels of tPA. After a Benjamini-Hochberg correction for multiple testing, the proportion of mediation by tPA did not remain significant (*p* = 0.120).

The sensitivity analysis on patients with pulmonary ARDS and the adjusted analysis exclusion of CCI and APACHE-IV as covariates showed similar results, but no significant mediation in the subgroup of pulmonary ARDS (see Additional file 1: Table S10–S14 and Additional file 1: Table S15–S18). The sensitivity analysis with age as a continuous variable also showed a similar result, with statistically significant partial mediation of 9 [1 to 26] % (*p* = 0.027) by tPA after adjustment for confounders (see Additional file 1: Table S19–21).

Last, the subgroup analysis of 350 patients (119 young adults, 110 middle-aged adults, and 121 elderly) with a sample at a later timepoint showed that compared to young patients, elderly patients had again lower plasma levels of IL-6, IL-8, IL-10, INF-γ, fractalkine, ICAM-1, and MMP8 (Additional file 1: Figure S3), while plasma levels of PF4 and tPA were not found to be higher in elderly.

## Discussion

In this prospective cohort of ARDS patients, a clear association was found between advanced age and higher mortality, which remained after adjustment for potential confounders including comorbidity and severity of illness scores. A mediation analysis showed only moderate (10%) mediation of this association by tPA. In contrast to the tested hypothesis, a comparison of the inflammatory mediators and endothelial activation markers revealed *lower* instead of *higher* plasma levels in elderly compared to young patients. Advanced age was only independently associated with increased plasma levels of tPA and decreased plasma levels of fractalkine and E-selectin.

The importance of the association between age and outcome in critically ill patients has been recognized over the past decades [4, 5]. Understanding of the underlying pathophysiological mechanisms explaining this association may provide novel therapeutic targets. To our best knowledge, this is the first study in humans that investigates age-related differences in systemic host response during ARDS as a potential mediator of the increased mortality in elderly by using a mediation analysis.

Although tPA was the only marker showing mediation and the higher levels of tPA in elderly explained just 10% of the association between age and outcome, this finding deserves attention. Increased levels of tPA were strongly associated with mortality and were significantly higher in elderly. Moreover, PAI-1, the endogenous inhibitor of tPA, tended to be lower in elderly, thereby shifting the balance tPA/PAI-1 towards fibrinolysis. Interesting in this context, recent studies have shown that both fibrinolysis shutdown and hyper-fibrinolysis are associated with increased mortality after acute injury [44, 45]. Besides its contribution to fibrinolysis, tPA has been shown to play a role in various other mechanisms involved in the pathophysiology of ARDS, such as the turnover of extracellular matrix components and extravasation of neutrophils [46, 47] [48]. Taking together, tPA could be a potential “modifiable mediator” of interest. However, it must be stressed that we performed multiple mediation models and the partial mediation of tPA did not remain significant after correction for multiple testing. Thus, this effect maybe a false discovery due to a type-1 error. Therefore, it is important that future studies confirm a role of tPA in the association between age and outcome.

The current data showed no evidence of mediation though an enhanced inflammatory mediator response or endothelial activation. Instead, the elderly had lower plasma levels of these biomarkers compared to young patients. Lower levels of systemic inflammatory markers in critically ill elderly have previously been described and could indicate an inadequate host response (so-called immunosenescence) [17]. Nevertheless, so far no association between immunosenescence and an adverse outcome of critically ill elderly has been described [17]. Of note, the mediation analysis showed that the lower biomarker levels found in elderly were not explanatory for the observed increased mortality in this age group. However, as multiple aspects of immunosenescence are known to be mediated via the cellular immune response, which was not included in the current analysis, a role of immunosenescence cannot be ruled out [49]. In contrast to immunosenescence, the lower levels of inflammatory markers in elderly may also reflect differences in the precipitating injury. But, beside transplantation, we found no significant differences in predisposing factors between the age groups. Furthermore, it is important to realize that the host response is a dynamic process. We assessed the host response at the onset of ARDS, i.e., in the early phase of ARDS, while the differences in mortality seem to occur much later (e.g., 90-day mortality and 1-year mortality). Potential age-dependent differences in the host response at a later time point could exist and may also explain outcome differences. Studies have suggested that a prolonged inflammatory response is associated with an adverse outcome [50, 51]. Moreover, there is evidence of age-dependent temporal differences in the host response in critically ill patients (e.g., a hyper-inflammatory response during the later phase of disease and a prolonged inflammation in elderly) [17, 20, 52–56]. The sensitivity analysis of a subset of patients with a sample at a later timepoint did not show any evidence for such prolonged inflammation in elderly. Nevertheless, serial sampling should be included in the design of future studies to assess the presence and implication of time-related differences and their relation to outcome in elderly ARDS patients. In addition, age-related differences in mechanisms involving resolution and repair of damage may also be important [57], especially, as the major differences between younger and elderly patients appear to concern the long-term outcome. Unfortunately, these mechanisms were not studied here due to the restriction in samples available for this analysis.

Despite the unambiguous evidence from various animal models of acute injury, clinical studies—including the current study—failed to detect a robust age-related difference in the systemic host response system that can explain the adverse outcome of elderly with ARDS (see Additional file 1: Table S23). Instead, the data from these clinical studies indicates that elderly ARDS patients die of other reasons than an overwhelming systemic host response. An important contributor to outcome in elderly may be “frailty,” which is characterized by the vulnerability to an acute stressor [58], and its interaction with physiological reserve, i.e., the ability to maintain and restore vital functions [59, 60]. Therefore, the relation between the biomarker levels and outcome may differ according to age. This could have important consequences for the use of biomarkers and biomarker profiles for prognostication, prediction, and selection of patients for clinical trials.

One strength of this study is its prospective design and the use of a broad range of biomarkers characterizing the three main pathways involved in the host response to injury during ARDS. However, study limitations also need to be considered. First, age-groups cut-offs were based on age-tertiles of the total ICU population. These chronological boundaries may not reflect the accurate biological stages of aging [61], although the sensitivity analysis with age as a continuous variable showed similar results. Second, the current study is a secondary analysis of a subset of patients in the parental MARS project, performed on the available dataset. Thus, the absence of statistical significance may be due to a lack of power. In addition, the included ARDS patients formed a heterogeneous patient population. This heterogeneity may have increased the variation in the host response, thereby limiting the ability to detect subtle influences of age-related differences on outcome. However, the subgroup analysis of patients with pulmonary ARDS did not change the results. Moreover, our cohort is relatively large compared to previous studies (see Additional file 1: Table S23), which implies more precision of the estimates. Strikingly, some of the biomarkers showed significant “negative mediation.” This could be a statistical artifact (e.g., unidentified confounding or a result of multiple testing), but a biological cause of this so-called inconsistent mediation (i.e., a suppressor effect) cannot be excluded [62]. Still, because negative mediation can per definition not explain the increased mortality in elderly further exploration of this negative mediation is beyond the scope of our study. Third, despite the strict protocol for timely handling and storage of the daily left-over EDTA, the use of discarded blood by itself could have influenced the biomarker level. It should also be noted, though, that all samples were handled in the same way and irrespective of the age of patients. Fourth, we investigated only the systemic biomarkers. The pulmonary response might be different from the systemic response and more representative for mechanisms contributing to the outcome of ARDS [16]. In addition, it was not possible to determine indices of the activation of the cellular immune response as we had only daily left-over EDTA anti-coagulated plasma samples available for analysis. This is another limitation of the study because there is growing evidence that the cellular immune response plays an important role in the regulation of the inflammatory response and the progression of ARDS [63]. Moreover, instead of looking at single markers, we may have to use a broader approach like genomics and proteomics or combine biomarkers into profiles—by methods like cluster or class analysis—to identify the biological subtypes, so-called “phenotypes” [29, 64]. It has been shown that clinical outcomes and responses to treatment depend on the phenotype of the patient [64]. Whether or not such phenotypes are depending on age or differ between different age groups need further attention.

Finally, the lack of information on the causes and circumstances of death is an important limitation of our study. We assessed the host response at an early stage of disease while death occurs much later in time. Consequentially, patients may have died of other factors than the progression of their initial disease. Previous studies have indicated that age is one of the determinants in end-of-life decisions [65]. Age-related differences in the limitation on life-sustaining measures may have introduced selection bias, which can prevent the detection of a biological relation between the host response and outcome. To minimize this influence of age-related decision-making patients with a limitation on life-sustaining measures at admission were excluded. Furthermore, the available data from patients that died in the ICU showed high respiratory and cardiovascular SOFA scores independent of age, and no age-related differences in the limitation on life-sustaining measures on the day the patients died. Still, selection bias may have influenced the outcome of the study. Last, 92 of the 818 patients with ARDS had to be excluded because there was no blood sample available at the onset of ARDS, this may have introduced selection bias. However, there was no difference in the proportion of missing samples among the age groups.

## Conclusion

In this cohort of ARDS patients, the observed association between advanced age and increased mortality was unrelated to alterations in systemic inflammation and endothelial cell activation. Only tPA was found to be a significant partial mediator of the association between advanced age and increased mortality, a mediation effect that disappeared after correction for multiple testing.

## Supplementary information

**Additional file 1.** Online supplement data and Tables S1–S23 and Figures S1–S3

## Data Availability

The data that support the findings of this study are available from the MARS consortium, but restrictions apply to the availability of these data, which were used under license for the current study, and so are not publicly available. Data are however available from the authors upon reasonable request and with permission of MARS consortium.

## Abbreviations

ACME: Average causal mediation effect
ADE: Average direct effect
ANG: Angiopoetin
APACHE: Acute physiology and chronic health evaluation
ARDS: Acute respiratory distress syndrome
CI: Confidence interval
ICAM: Intracellular adhesion molecule
IL: Interleukin
INF-γ: Interferon-gamma
IQR: Interquartile range
MMP-8: Matrix metalloproteinase-8
OR: Odds ratio
PAI-1: Plasminogen activator inhibitor-1
PBW: Predicted body weight
PEEP: Positive end-expiratory pressure
PF4: Platelet factor 4
SOFA: Sequential organ failure assessment
TIMP-1: Metallopeptidase inhibitor-1
TNF-α: Tumor necrosis factor alpha
tPA: Tissue plasminogen activator

## References

[1] Ely EW, Wheeler AP, Thompson BT (2002). Recovery rate and prognosis in older persons who develop acute lung injury and the acute respiratory distress syndrome. Ann Intern Med.

[2] Manzano F, Yuste E, Colmenero M (2005). Incidence of acute respiratory distress syndrome and its relation to age. J Crit Care.

[3] Schouten LRA, Schultz MJ, van Kaam AH (2015). Association between maturation and aging and pulmonary responses in animal models of lung injury: a systematic review. Anesthesiology.

[4] Villar J, Ambrós A, Soler JA (2016). Age, PaO_2_/FIO_2_, and plateau pressure score: a proposal for a simple outcome score in patients with the acute respiratory distress syndrome. Crit Care Med.

[5] Herridge MS, Chu LM, Matte A (2016). The RECOVER program: disability risk groups and 1-year outcome after 7 or more days of mechanical ventilation. Am J Respir Crit Care Med.

[6] Wunsch H, Linde-Zwirble WT, Angus DC (2010). The epidemiology of mechanical ventilation use in the United States. Crit Care Med.

[7] Montgomery RR, Shaw AC (2015). Paradoxical changes in innate immunity in aging: recent progress and new directions. J Leukoc Biol.

[8] Shaw AC, Goldstein DR, Montgomery RR (2013). Age-dependent dysregulation of innate immunity. Nat Rev Immunol.

[9] Ferrucci L, Harris TB, Guralnik JM (1999). Serum IL-6 level and the development of disability in older persons. J Am Geriatr Soc.

[10] Cohen HJ, Harris T, Pieper CF (2003). Coagulation and activation of inflammatory pathways in the development of functional decline and mortality in the elderly. Am J Med.

[11] Niwa Y, Kasama T, Miyachi Y, Kanoh T (1989). Neutrophil chemotaxis, phagocytosis and parameters of reactive oxygen species in human aging: cross-sectional and longitudinal studies. Life Sci.

[12] Butcher SK, Chahal H, Nayak L (2001). Senescence in innate immune responses: reduced neutrophil phagocytic capacity and CD16 expression in elderly humans. J Leukoc Biol.

[13] Oakley R, Tharakan B (2014). Vascular hyperpermeability and aging. Aging Dis.

[14] Franceschi C, Bonafè M, Valensin S (2000). Inflamm-aging. An evolutionary perspective on immunosenescence. Ann N Y Acad Sci.

[15] Saito H, Sherwood ER, Varma TK, Evers BM (2003). Effects of aging on mortality, hypothermia, and cytokine induction in mice with endotoxemia or sepsis. Mech Ageing Dev.

[16] Turnbull IR, Clark AT, Stromberg PE (2009). Effects of aging on the immunopathologic response to sepsis. Crit Care Med.

[17] Stanojcic M, Chen P, Xiu F, Jeschke MG (2016). Impaired immune response in elderly burn patients: new insights into the immune-senescence phenotype. Ann Surg.

[18] Ginde AA, Blatchford PJ, Trzeciak S (2014). Age-related differences in biomarkers of acute inflammation during hospitalization for sepsis. Shock.

[19] Marik PE, Zaloga GP, NORASEPT II Study Investigators. North American Sepsis Trial II (2001). The effect of aging on circulating levels of proinflammatory cytokines during septic shock. Norasept II Study Investigators. J Am Geriatr Soc.

[20] Kale S, Yende S, Kong L (2010). The effects of age on inflammatory and coagulation-fibrinolysis response in patients hospitalized for pneumonia. PLoS One.

[21] Kelly E, MacRedmond RE, Cullen G (2009). Community-acquired pneumonia in older patients: does age influence systemic cytokine levels in community-acquired pneumonia?. Respirology.

[22] Glynn P, Coakley R, Kilgallen I, O’Neill S (1999). Neutrophil CD11b and soluble ICAM-1 and E-selectin in community acquired pneumonia. Eur Respir J.

[23] Boldt J, Müller M, Heesen M (1997). Does age influence circulating adhesion molecules in the critically ill?. Crit Care Med.

[24] Bos Lieuwe D., Schouten Laura R., Schultz Marcus J. (2016). Promising but still uncertain steps towards better prediction of functional outcome in ICU patients. Journal of Thoracic Disease.

[25] Klein Klouwenberg PMC, Frencken JF, Kuipers S (2017). Incidence, predictors, and outcomes of new-onset atrial fibrillation in critically ill patients with sepsis. A cohort study. Am J Respir Crit Care Med.

[26] Klein Klouwenberg PMC, van Mourik MSM, Ong DSY (2014). Electronic implementation of a novel surveillance paradigm for ventilator-associated events. Feasibility and validation. Am J Respir Crit Care Med.

[27] Geboers DGPJ, de Beer FM, Tuip-de Boer AM (2015). Plasma suPAR as a prognostic biological marker for ICU mortality in ARDS patients. Intensive Care Med.

[28] van Vught LA, Wiewel MA, Hoogendijk AJ (2017). The host response in patients with sepsis developing intensive care unit-acquired secondary infections. Am J Respir Crit Care Med.

[29] Bos LD, Schouten LR, van Vught LA (2017). Identification and validation of distinct biological phenotypes in patients with acute respiratory distress syndrome by cluster analysis. Thorax.

[30] Hoogendijk AJ, Wiewel M A, van Vught L A, et al (2015) Plasma fractalkine is a sustained marker of disease severity and outcome in sepsis patients. Crit Care 19:412. doi: 10.1186/s13054-015-1125-0. 10.1186/s13054-015-1125-0 PMC4658804 26603530

[31] van Vught LA, Scicluna BP, Hoogendijk AJ (2016). Association of diabetes and diabetes treatment with the host response in critically ill sepsis patients. Crit Care.

[32] Boshuizen M, Leopold JH, Zakharkina T (2015). Levels of cytokines in broncho-alveolar lavage fluid, but not in plasma, are associated with levels of markers of lipid peroxidation in breath of ventilated ICU patients. J Breath Res.

[33] Frencken JF, van Vught LA, Peelen LM (2017). An unbalanced inflammatory cytokine response is not associated with mortality following Sepsis: a prospective cohort study. Crit Care Med.

[34] Wiewel MA, de Stoppelaar SF, van Vught LA (2016). Chronic antiplatelet therapy is not associated with alterations in the presentation, outcome, or host response biomarkers during sepsis: a propensity-matched analysis. Intensive Care Med.

[35] Bernard GR, Artigas A, Brigham KL (1994). Report of the American-European Consensus Conference on acute respiratory distress syndrome: definitions, mechanisms, relevant outcomes, and clinical trial coordination. J Crit Care.

[36] Ranieri VM, Rubenfeld GD, Thompson BT (2012). Acute respiratory distress syndrome: the Berlin definition. JAMA.

[37] O’Shea JJ, Murray PJ (2008). Cytokine signaling modules in inflammatory responses. Immunity.

[38] Yang XP, Mattagajasingh S, Su S (2007). Fractalkine upregulates intercellular adhesion molecule-1 in endothelial cells through CX3CR1 and the Jak Stat5 pathway. Circ Res.

[39] Levi M, van der Poll T, Büller HR (2004). Bidirectional relation between inflammation and coagulation. Circulation.

[40] Song Y, Huang YT, Song Y (2015). Birthweight, mediating biomarkers and the development of type 2 diabetes later in life: a prospective study of multi-ethnic women. Diabetologia.

[41] Beulens JWJ, van der Schouw YT, Moons KGM (2013). Estimating the mediating effect of different biomarkers on the relation of alcohol consumption with the risk of type 2 diabetes. Ann Epidemiol.

[42] Valeri L, Vanderweele TJ (2013). Mediation analysis allowing for exposure-mediator interactions and causal interpretation: theoretical assumptions and implementation with SAS and SPSS macros. Psychol Methods.

[43] Tingley D, Yamamoto T, Hirose K (2014). Mediation: R package for causal mediation analysis. J Stat Softw.

[44] Moore HB, Moore EE, Gonzalez E (2014). Hyperfibrinolysis, physiologic fibrinolysis, and fibrinolysis shutdown: the spectrum of postinjury fibrinolysis and relevance to antifibrinolytic therapy. J Trauma Acute Care Surg.

[45] Moore HB, Moore EE, Liras IN (2016). Acute fibrinolysis shutdown after injury occurs frequently and increases mortality: a multicenter evaluation of 2,540 severely injured patients. J Am Coll Surg.

[46] Montrucchio G, Lupia E, De Martino A (1996). Plasmin promotes an endothelium-dependent adhesion of neutrophils. Circulation.

[47] Baramova EN, Bajou K, Remacle A (1997). Involvement of PA/plasmin system in the processing of pro-MMP-9 and in the second step of pro-MMP-2 activation. FEBS Lett.

[48] Zhao Y, Sharma AK, LaPar DJ (2011). Depletion of tissue plasminogen activator attenuates lung ischemia-reperfusion injury via inhibition of neutrophil extravasation. Am J Physiol Lung Cell Mol Physiol.

[49] Fulop T, Larbi A, Dupuis G (2017). Immunosenescence and inflamm-aging as two sides of the same coin: friends or foes?. Front Immunol.

[50] Narute P, Seam N, Tropea M (2017). Temporal changes in microrna expression in blood leukocytes from patients with the acute respiratory distress syndrome. Shock.

[51] Meduri GU, Headley S, Kohler G (1995). Persistent elevation of inflammatory cytokines predicts a poor outcome in ARDS: plasma IL-1β and IL-6 levels are consistent and efficient predictors of outcome over time. Chest.

[52] Yokota Y, Wakai Y, Mine Y (1988). Degradation of host defenses against respiratory tract infection by Klebsiella pneumoniae in aged mice. Infect Immun.

[53] Wen J, Li C-M, Gu L (2014). Aging reduces the expression of lung CINC and MCP-1 mRNA in a *P. aeruginosa* rat model of infection. Inflammation.

[54] Mares CA, Ojeda SS, Li Q (2010). Aged mice display an altered pulmonary host response to Francisella tularensis live vaccine strain (LVS) infections. Exp Gerontol.

[55] Mares CA, Sharma J, Ojeda SS (2010). Attenuated response of aged mice to respiratory Francisella novicida is characterized by reduced cell death and absence of subsequent hypercytokinemia. PLoS One.

[56] Pinheiro da Silva F, Zampieri FG, Barbeiro DF (2013). Septic shock in older people: a prospective cohort study. Immun Ageing.

[57] López-Otín C, Blasco MA, Partridge L (2013). The hallmarks of aging. Cell.

[58] McDermid RC, Stelfox HT, Bagshaw SM (2011). Frailty in the critically ill: a novel concept. Crit Care.

[59] Linge HM, Lee JY, Ochani K (2015). Age influences inflammatory responses, hemodynamics, and cardiac proteasome activation during acute lung injury. Exp Lung Res.

[60] Rosas GO, Zieman SJ, Donabedian M (2001). Augmented age-associated innate immune responses contribute to negative inotropic and lusitropic effects of lipopolysaccharide and interferon gamma. J Mol Cell Cardiol.

[61] Milbrandt EB, Eldadah B, Nayfield S (2010). Toward an integrated research agenda for critical illness in aging. Am J Respir Crit Care Med.

[62] MacKinnon DP, Krull JL, Lockwood CM (2000). Equivalence of the mediation, confounding and suppression effect. Prev Sci.

[63] Lin S, Wu H, Wang C (2018). Regulatory T cells and acute lung injury: cytokines, uncontrolled inflammation, and therapeutic implications. Front Immunol.

[64] Calfee CS, Delucchi K, Parsons PE (2014). Subphenotypes in acute respiratory distress syndrome: latent class analysis of data from two randomised controlled trials. Lancet Respir Med.

[65] Joynt GM, Gomersall CD, Tan P (2001). Prospective evaluation of patients refused admission to an intensive care unit: triage, futility and outcome. Intensive Care Med.

