# Supplementary material for: Increased mortality in elderly patients with acute respiratory distress syndrome is not explained by host response
Source: Intensive Care Med Exp. 2019 Oct 29;7:58. doi: 10.1186/s40635-019-0270-1 (PMC6820655; doi:10.1186/s40635-019-0270-1)
Supplement: Supplementary file 1 — Additional file 1. Online supplement data and Tables S1–S23 and Figures S1–S3 [file 40635_2019_270_MOESM1_ESM.docx]

Supplement to:

**Increased Mortality in Elderly Patients with Acute Respiratory Distress Syndrome is not Explained by Host Response**

Laura RA Schouten, MD^1,2,3^, Lieuwe DJ Bos, MD PhD^2,3^, Prof Serpa Neto A, MD PhD^2,4^, Lonneke A van Vught, MD PhD^5^, Maryse A Wiewel, MD PhD^5^, Arie J Hoogendijk, MD PhD^5^, Prof Marc J Bonten, MD PhD^6^, Olaf L Cremer, MD PhD^7^, Janneke Horn, MD PhD^2,3^, Prof Tom van der Poll, MD PhD^5^, Prof Marcus J Schultz, MD PhD^2,3,8^, Roelie M Wösten–van Asperen, MD PhD^9^, the MARS consortium*

**eMethods**

**Methods** – Clinical data collection

**Methods** – Sample collection and assays

**Table S1** – Number of values under the detection limit

**Methods** – Explanation of the mediation analysis

**Methods** – Handling missing data for the multiple regression models

**Table S2** – Missing data of the values included in the mediation model.

**eResults**

**Table S3** – Comorbidities, predisposing factors, oxygenation and laboratory parameters at onset of ARDS

**Figure S1**– Kaplan-Meier plots of survival in ARDS patient

**Table S4** – Outcome of ARDS patients

**Table S5** – Association between age and mortality in ARDS patients

**Table S6** – Sequential organ failure scores and information on the restriction on care at the day of death from patients that died on the ICU.

**Figure S2** – Biomarker levels stratified by age-groups

**Table S7** – Association between systemic biomarkers – 90 days mortality in ARDS patients (*Mediation step 2*)

**Table S8** – Mediation pathway analysis – age, systemic biomarkers and mortality, univariate model *(Mediation step 4)*

**Table S9** – Mediation pathway analysis – age, systemic biomarkers and mortality, multiple regression models *(Mediation step 4)*

**Sensitivity analyses**:

***Subgroup analysis of patient with pulmonary ARDS:***

**Table S10** Covariates of patients with pulmonary ARDS

**Table S11** Association between age and mortality in patients with pulmonary ARDS (*Mediation step 1*)

**Table S12** Association between systemic biomarkers – 90 days mortality in pulmonary–patients with pulmonary ARDS (*Mediation step 2*)

**Table S13** Association between age and biomarker levels of patients with pulmonary ARDS *(Mediation Step 3)*

**Table S14**. Mediation pathway analysis – age, systemic biomarkers and mortality, univariate and multiple regression models of patients with pulmonary ARDS *(Mediation step 4)*

***Adjusted analysis without CCI and comorbidity as covariates***

**Table S15** Association between age and mortality without CCI and comorbidity as covariates (*Mediation step 1*)

**Table S16** Association between systemic biomarkers – 90 days mortality in ARDS patients without CCI and comorbidity as covariates (*Mediation step 2*)

**Table S17** Association between age and biomarker levels in ARDS patients without CCI and comorbidity as covariates *(Mediation Step 3)*

**Table S18**. Mediation pathway analysis – age, systemic biomarkers and mortality, multiple regression models of patients ARDS without CCI and comorbidity as covariates *(Mediation step 4)*

***Sensitivity analysis with age as a continuous variable***

**Table S19** Association between age (continuous variable) and mortality (*Mediation step 1*)

**Table** **S20** Association between age (continuous variable) and inflammatory marker levels in ARDS patients *(Mediation Step 3)*

**Table S21** Mediation pathway analysis – age (continues variable), systemic biomarkers and mortality, univariate and multiple regression models *(Mediation step 4)*

***Exploration of age-related temporal differences***

**Table S22** Covariates of patients with a sample at a later timepoint.

**Figure S3.** Biomarker levels at a later timepoint, stratified by age-groups

**eBackground/Discussion**

**Table S23** – Results of a literature review

**eMETHODS**

**Clinical data collection**

The following data was collected prospectively from all patients: demographics, chronic co–morbidities, severity of illness score (Acute Physiology and Chronic Health Evaluation (APACHE)–IV score)[1], Sepsis–related Organ Failure Assessment (SOFA) score, predisposing factors for ARDS, presence of kidney injury during admission and ventilator settings, oxygenation parameters and clinical laboratory parameters at diagnosis of ARDS. As the APACHE–IV score contains age as contributing variable to the total score, we also re-calculated an APACHE–IV score without the contribution of age (APACHE–IV Score adjusted for age) by subtracting the point awarded based on age from the total APACHE–IV score. Based on the collected data, we calculate the Lung Injury Prediction Score (LIPS) and the Charlson Comorbidity Index (CCI)[2, 3]. Based on the predisposing factors patients were classified as having a direct–(i.e pneumonia, aspiration, inhalation trauma, near drowning, or another pulmonary challenge) or an indirect-hit for ARDS (i.e. none pulmonary systemic inflammatory response syndrome– or sepsis–associated challenge). In addition, the for all patients we assessed the vital status in the GBA (the government registration of persons) 1 year after the initial admission to the intensive care unit. In case a patient was deceased, we recorded the specific date of death, therefore there is no censored mortality data. For the subset of patients that died on the ICU, the occurrence of death was recorded prospectively and the SOFA score and the restriction on care were recorded at the day of death.

**Sample collection and assays**

Daily - on admission and at 6 AM thereafter - left–over plasma harvested from blood obtained for regular patient care was kept at 4^°^C and stored at –80^°^C within 4 hours[4]. Biomarker measurements for the current study were performed in EDTA anti–coagulated plasma at onset of ARDS. All biomarkers were measured with customized multiplex kits. Interleukin (IL)–6, IL–8, IL–10, IL–1β, tumor necrosis factor alpha (TNF–α), interferon gamma (INF–γ), E–selectin, P–selectin and fractalkine were measured using a cytometric bead array (CBA) Flex Set multiplex assay according to the instructions from the manufacturer (BD Biosciences, San Jose, CA). Protein C, plasminogen activator inhibitor (PAI)–1, antithrombin (AT), intracellular adhesion molecule (ICAM)–1, matrix metalloproteinase (MMP)–8, metallopeptidase inhibitor (TIMP)–1, angiopoetin (ANG)–1, ANG–2, platelet factor 4 (PF4) (all R&D systems, Abingdon, UK), D–dimer and tissue plasminogen activator (tPA) (both Procartaplex, eBioscience, San Diego, CA), were measured with Luminex according to the manufacturer instructions (BioRad, Hercules, CA, USA). The measurements were performed batchwise. Values under the lower limit of detection were set at the lowest limit of detection (Table S1).

**Table S1 Number of values under the detection limit**.

| **Biomarkers** | **Samples under the LODD**  **n / total n (%)** | |
| --- | --- | --- |
| IL-6 | 0/618 | (0) |
| Il-8 | 0/618 | (0) |
| IL-10 | 13/618 | (2) |
| IL–1β | 242/618 | (39) |
| TNF–α | 395/618 | (64) |
| INF–γ | 286/618 | (46) |
| ICAM–1 | 0/618 | (0) |
| MMP–8 | 0/618 | (0) |
| TIMP–1 | 0/618 | (0) |
| Fractalkine | 95/618 | (15) |
| E–selectin | 0/618 | (0) |
| P–selectin | 0/618 | (0) |
| ANG2/ANG1 | 0/618 | (0) |
| PF4 | 0/618 | (0) |
| Protein–C | 0/618 | (0) |
| PAI–1 | 0/618 | (0) |
| AT | 0/618 | (0) |
| D–dimer | 0/618 | (0) |
| tPA | 0/618 | (0) |

LODD, lower limit of detection

**Explanation of the mediation analysis** [5–12]

A mediation analysis is a stepwise statistical approach to investigate the mechanisms explaining an association between an independent variable (X) and a dependent variable (Y), thus investigating potential causal pathways mediating the association between X and Y. In the present study we hypothesized that advanced age (X) is associated with increased mortality (Y), and this association is mediated though an enhanced host response (M), reflected by increased plasma concentrations of biomarkers (e.g. biomarkers of inflammation, endothelial activation and coagulation). Of note, this mediation analysis is a two-tailed analysis, it can also detect mediation via a decreased host response (M) (i.e. lower plasma concentrations of biomarkers).

We tested if the age-group (X) had a *direct* impact in mortality (Y) or, whether there is an *indirect* effect of age on mortality via a change (either an increase or decrease) in biomarker levels. To be a mediator (M), the biomarker must be included in the causal pathway between age and mortality (Figure 1). Thus, the biomarker level must be effected by age and subsequently this age- induced change in the biomarker level must effect mortality. Importantly, this indirect effect must be independent of the main effect of age on mortality. If the association between age and outcome is completely explained by variation in biomarker concentration, then there is so-called *complete mediation*. However, complete mediation is not obligated to demonstrate mediation. Actually, based on biological plausibility a single biomarker is more likely to be a *partial mediator,* thus the indirect effect explains only a fraction of the total effect observed between X and Y. A priori we considered < 5% as a small, 5–20% as a moderate and > 20% as a large proportion of mediation.

A mediation analysis is a stepwise pathway analysis using a sequence of univariate and multiple regression models. The first step in the analysis is to test the main association between age (X) and mortality (Y), the so-called *direct effect* (coefficient C, reflecting the average direct effect (ADE)) (*Figure 1- mediation step 1*). This was calculated through univariate and multiple logistic regression analysis. In the second step, we tested if a change in a mediator level is associated with a change in outcome, thus whether the biomarker concentration was associated with mortality (*Figure 1 – mediation step 2*). For this step we used separate logistic regression models per biomarker (coefficient B). In the third step (*Figure 1 – mediation step 3*), we tested if age is associated with t biomarker levels. Accordingly, we used linear regression to model the association between age and each biomarker (coefficient A). In a fourth step (*Figure 1 – mediation step 4*), we used a procedure that is equivalent to the Sobel test. In this step, we tested if biomarker levels significantly impact the mortality when included in a multiple regression model together with age (and potential confounders: ethnic background, gender, admission type, readmission, direct-hit for ARDS, Charlson Comorbidity Index, APACHE IV adjusted for age, immunodeficiency, tidal volume (VT) per predicted body weight, and positive end–expiratory pressure). Thus, whether the coefficient C’ (representing the direct effect of age on mortality after multiple adjustment) becomes weaker after accounting for the mediating effect (indirect path "A*B", average causal mediation effect (ACME)). In case of a complete mediation, ADE will become zero and non-significant, while ACME is equal to the total effect and is significant. The proportion of mediation was expressed as the ratio between ACME:ADE. Mediation was only considered relevant if the point estimate of the mediated effect was in the same direction as the total effect because ‘negative mediation’ can per definition not contribute to the increased mortality. Importantly to note, if an increase in age would be associated with a decrease in the biomarker levels (pathway A = negative point estimate), and a decrease in the biomarker levels is associated with higher mortality (pathway B = negative point estimate), the point estimate of the mediated effect (ACME) would still be positive (–A * –B = positive point estimate).

Interaction between the independent variable and the mediator was tested, none of the biomarkers showed significant interaction with the independent variable. For this mediation analysis, biomarkers were log transformed to obtain normally distributed variables and young–adults were defined as the reference group.


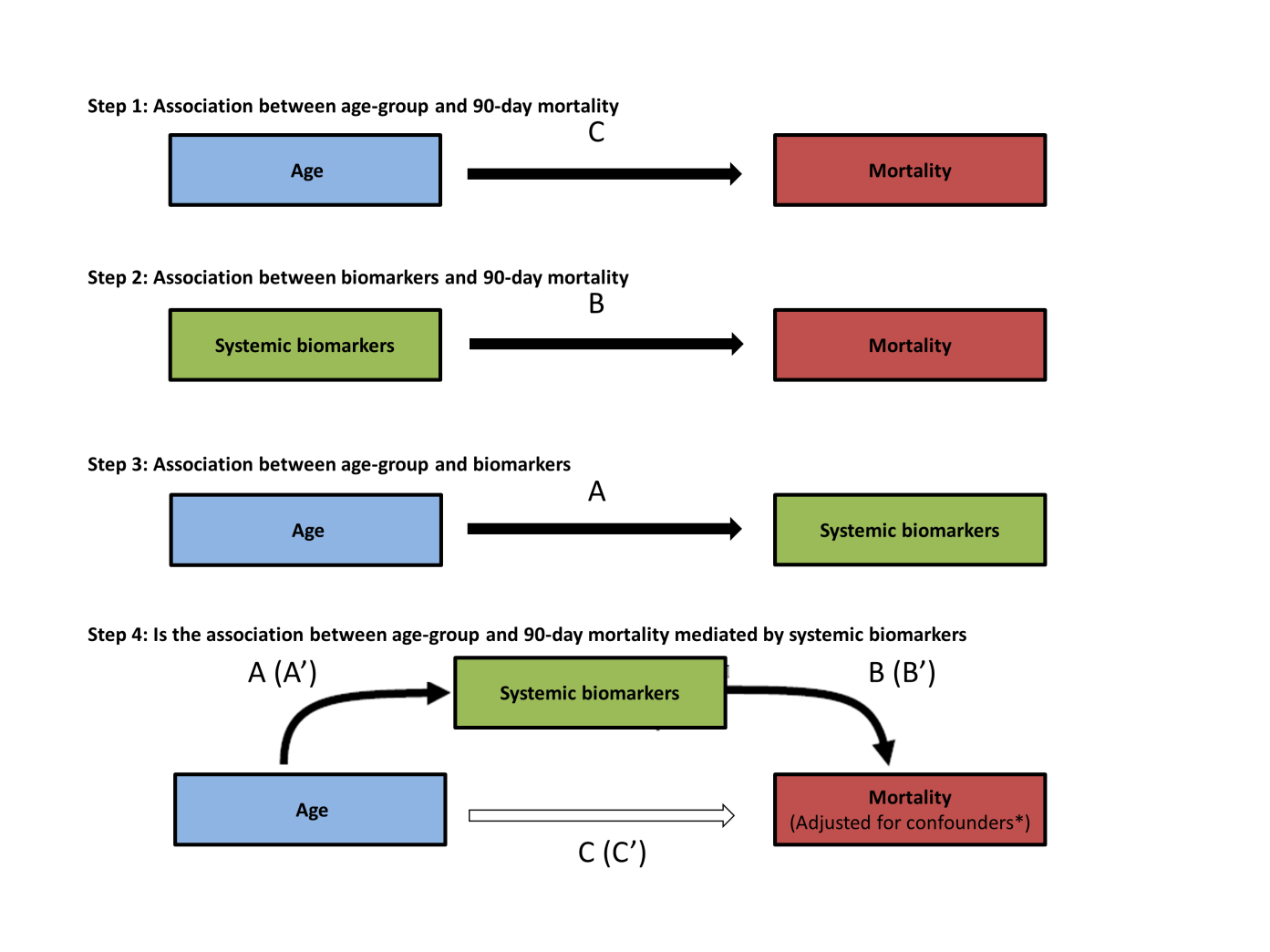


Pathway analysis: stepwise mediation analysis assessing whether the association between age and mortality is mediated by age–dependent differences in biomarker levels. *Adjusted for: ethnic background, gender, admission type, readmission, direct–hit for ARDS, Charlson Comorbidity Index, APACHE–IV score adjusted for age, immunodeficiency, tidal volume per predicted body weight, positive end expiratory pressure. C = average direct effect (ADE); A*B = average causal mediation effect (ACME). A’, B’ and C’ – adjusted for confounders.

**Handling missing data for the multiple regression models**

After initial data-collection, missing data or inconsistencies were checked by queries and if possible added manually by the research team. In the multiple regression analysis, multiple imputation chained equations were used to replace missing data^13^. There were no missing values for the covariates gender, readmission and Charlson Comorbidity Index (Table E2). Admission type was missing in 1 (0%) young patient, 1 (0%) middle-aged patient and 0 (0%) elderly patients. Patient ethnic background was missing in 9 (4%) young patients, 6 (3%) middle-aged patients and 7 (4%) elderly patients. Type of insult was unknown in 1(0%) young patient, 2 (1%) middle-aged patients and 4 (2%) elderly patients. APACHE IV score adjusted for age was missing in 1 (0%) young patient, 1 (0%) middle-aged patient and 1 (0%) elderly patient. Immunodeficiency was missing in 11 (5 %) young patients, 11 (5%) middle-aged patients and 11 (6%) elderly patients. Tidal volume per predicted body weight was missing in 44 (21%) young patients, 46 (22%) middle aged patients, and 51 (26%) elderly patients. Positive end expiratory pressure was missing in 38 (18%) young patients, 42 (20%) middle-aged patients and 46 (23%) elderly patients.

**Table S2** Missing data of the values included in the mediation model.

|  | **Young adults**  **(n=209)** | | **Middle–aged adults**  **(n=213)** | | **Elderly**  **(n=196)** | |
| --- | --- | --- | --- | --- | --- | --- |
| **Covariates** |  |  |  |  |  |  |
| Race, n (%) | 9 | (4) | 6 | (3) | 7 | (4) |
| Gender, n (%) | 0 | (0) | 0 | (0) | 0 | (0) |
| Admission type, n (%) | 0 | (0) | 0 | (0) | 0 | (0) |
| Readmission, n (%) | 0 | (0) | 0 | (0) | 0 | (0) |
| Direct-hit of ARDSt, n (%) | 1 | (0) | 2 | (1) | 4 | (2) |
| CCI | 0 | (0) | 0 | (0) | 0 | (0) |
| Immune deficiency, n (%) | 11 | (5) | 11 | (5) | 11 | (6) |
| APACHE score, n (%) | 1 | (0) | 1 | (0) | 1 | (0) |
| Tidal volume per PBW, n (%) | 44 | (21) | 46 | (22) | 51 | (26) |
| PEEP, n (%) | 38 | (18) | 42 | (20) | 46 | (23) |

CCI, Charlson Comorbidity Index; APACHE, acute physiology and chronic health evaluation; PBW, predicted body weight; PEEP, positive end-expiratory pressure

**eRESULTS**

**Table S3** Comorbidities, predisposing factors, oxygenation and laboratory parameters at onset of ARDS

|  | **Young adults**  **(n=209)** | | **Middle–aged adults**  **(n=213)** | | **Elderly**  **(n=196)** | | **p–value** |
| --- | --- | --- | --- | --- | --- | --- | --- |
| **Comorbidities** |  |  |  |  |  |  |  |
| Cardiovascular disease, n/total n (%) | 23/209 | (11) | 57/213 | (27) | 75/196 | (38) | <0.001 |
| Chronic lung disease, n/total n (%) | 31/209 | (15) | 54/213 | (25) | 42/196 | (21) | 0.037 |
| Diabetes Mellitus, n/total n (%) | 19/209 | (9) | 31/213 | (15) | 42/196 | (21) | 0.002 |
| Malignancy, n/total n (%) | 38/209 | (18) | 48/213 | (23) | 37/196 | (19) | 0.492 |
| Chronic renal failure, n/total n (%) | 17/209 | (8) | 24/213 | (11) | 22/196 | (11) | 0.478 |
| Immune deficiency, n/total n (%) | 55/209 | (26) | 43/213 | (20) | 22/196 | (11) | 0.001 |
| Smoking (yes), n/total n (%) | 24/209 | (11) | 31/213 | (15) | 20/196 | (10) | 0.394 |
| **Predisposing factors^*^** |  |  |  |  |  |  |  |
| Pneumonia, n/total n (%) | 119/209 | (57) | 119/213 | (56) | 109/196 | (56) | 0.945 |
| Sepsis^†^, n/total n (%) | 46/209 | (22) | 56/213 | (26) | 42/196 | (21) | 0.461 |
| Aspiration, n/total n (%) | 21/209 | (10) | 21/213 | (10) | 18/196 | (9) | 0.960 |
| Pancreatitis, n/total n (%) | 2/209 | (1) | 4/213 | (2) | 7/196 | (4) | 0.187 |
| Transfusion, n/total n (%) | 15/209 | (7) | 15/213 | (7) | 8/196 | (4) | 0.351 |
| Major trauma surgery, n/total n (%) | 17/209 | (8) | 18/213 | (8) | 17/196 | (9) | 0.986 |
| Lung contusion, n/total n (%) | 18/209 | (9) | 13/213 | (6) | 12/196 | (6) | 0.528 |
| Neurological, n/total n (%) | 14/209 | (7) | 5/213 | (2) | 8/196 | (4) | 0.104 |
| Near drowning, n/total n (%) | 2/209 | (1) | 1/213 | (0) | 1/196 | (1) | 0.998 |
| Transplantation, n/total n (%) | 10/209 | (5) | 2/213 | (1) | 0/196 | (0) | <0.001 |
| Drug, n/total n (%) | 4/209 | (2) | 1/213 | (0) | 1/196 | (1) | 0.296 |
| Unknown, n/total n (%) | 1/209 | (0) | 2/213 | (1) | 4/196 | (2) | 0.320 |
| **Oxygenation at onset of ARDS** |  |  |  |  |  |  |  |
| FiO_2_^‡^, % median [IQR] | 50 | [40, 61] | 50 | [40, 70] | 50 | [40, 65] | 0.807 |
| paO_2_^‡^, mmHg median [IQR] | 86 | [70, 108] | 80 | [67, 101] | 83 | [70, 108] | 0.296 |
| paCO_2_^‡^,mmHg median [IQR] | 44 | [37, 52] | 43 | [38, 50] | 42 | [37, 53] | 0.856 |
| pH, median [IQR] | 7.39 | [7.31, 7.44] | 7.39 | [7.32, 7.44] | 7.39 | [7.32, 7.45] | 0.823 |
| **Laboratory parameters at onset of ARDS** |  |  |  |  |  |  |  |
| WBC count max ( x10^9/l), median [IQR] | 13.7 | [7.8, 19.7] | 13.4 | [9.45, 18.6] | 14.8 | [11.1, 20.7] | 0.025 |
| Platelets min (x10^9/l), median [IQR] | 137 | [67, 209] | 144 | [81, 227] | 186 | [117, 262] | <0.001 |
| Prothrombin time max (s), median [IQR] | 15 | [13, 18] | 15 | [12, 18] | 14 | [12, 17] | 0.481 |
| Lactate max (mmol/l), median [IQR] | 2.3 | [1.5, 4.6] | 2.5 | [1.6, 5.1] | 2.8 | [1.8, 5.0] | 0.517 |
| Creatinin max (μmol/l), median [IQR] | 97 | [68, 160] | 113 | [75, 198] | 115 | [83, 169] | 0.021 |
| C–reactive protein (mg/l), median [IQR] | 189 | [90, 288] | 206 | [114, 280] | 192 | [80, 284] | 0.327 |

* Predisposing factors are not exclusive, †Sepsis of a non-pulmonary cause, ‡at the highest alveolar-arterial-O_2_ gradient during the day of onset of ARDS, WBC, white blood cell count, max, maximum, min, minimum

**Figure S1** Kaplan-Meier plots of survival in ARDS patient


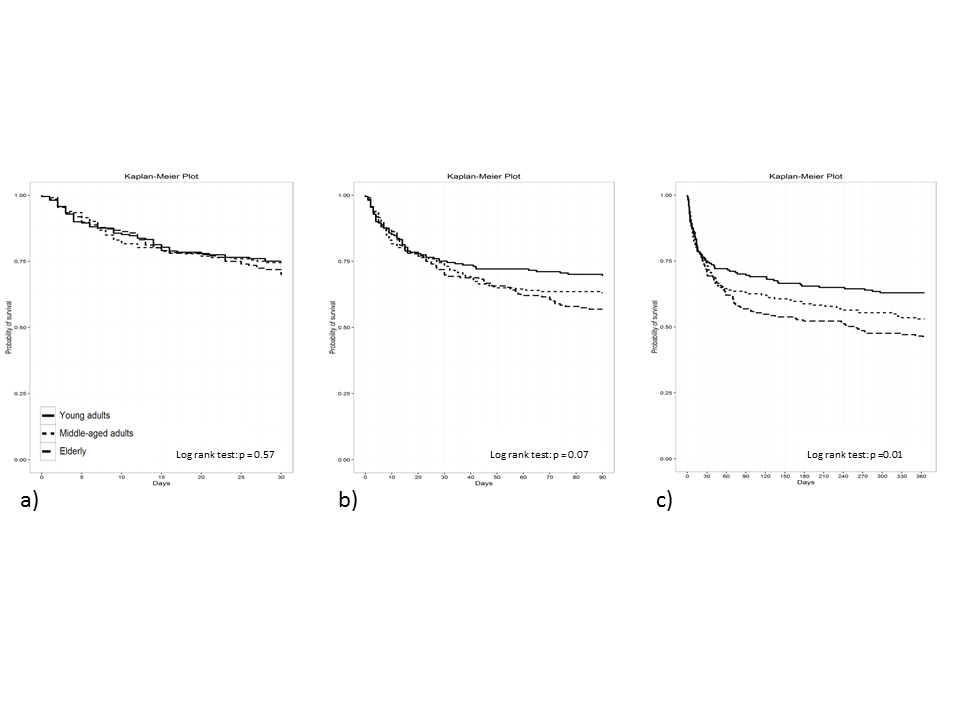


Survival curve of young, middle–aged, and elderly patients with ARDS. a) Kaplan–Meier plot of survival time up to 30 days after the onset of ARDS; log rank test p=0.568. b) 90 days after the onset of ARDS; log rank test p=0.072 and c) 1 year after the onset of ARDS; log rank test p<0.01.

**Table S4** Outcome of ARDS patients

|  | **Young adults**  **(n=209**) | | **Middle–aged adults**  **(n=213**) | | **Elderly**  **(n=196**) | | **p–value** |
| --- | --- | --- | --- | --- | --- | --- | --- |
|  |  |  |  |  |  |  |  |
| 90 days mortality*, n/total n (%) | 63/209 | (30) | 78/213 | (37) | 84/196 | (43) | 0.031 |
| ICU mortality, n/total n (%) | 44/209 | (21) | 48/213 | (23) | 46/196 | (23) | 0.831 |
| In–hospital mortality, n/total n (%) | 58/209 | (28) | 77/213 | (36) | 76/196 | (39) | 0.056 |
| 30 days mortality*, n/total n (%) | 52/209 | (25) | 56/213 | (26) | 59/196 | (30) | 0.484 |
| 1 year mortality*, n/total n (%) | 76/209 | (36) | 99/213 | (46) | 105/196 | (54) | 0.001 |
| Length of stay in hospital, median [IQR] | 30 | [14, 52] | 27 | [15, 49] | 27 | [16, 54] | 0.879 |
| Hospital free days and alive at day 90*, median [IQR] | 28 | [0, 61] | 12 | [0, 63] | 0 | [0, 56] | 0.010 |
| Length of stay in ICU, median [IQR] | 9 | [5, 17] | 9 | [5, 17] | 9 | [5, 14] | 0.903 |
| ICU free days and alive at day 30*, median [IQR] | 15 | [0, 23] | 15 | [0, 23] | 17 | [0, 23] | 0.947 |
| Ventilator free days and alive at day 28*, days, median [IQR] | 18 | [1, 25] | 20 | [1, 24] | 20 | [1, 24] | 0.782 |

* After the onset of ARDS. IQR, interquartile range, group differences between the age groups were tested by a chi-square-test. A p–value < 0.05 was considered as statistically significant.

**Table S5** Association between age and mortality in ARDS patients

|  | **Crude odds ratio**  **[95% CI]** | **p–value** | **Adjusted odds ratio**  **[95% CI]*** | **p–value** |
| --- | --- | --- | --- | --- |
| **ICU mortality** |  |  |  |  |
| **Young adults** | Reference |  |  |  |
| **Middle**–**aged adults** | 1.1 [0.7, 1.7] | 0.712 | 1.2 [0.7, 1.9] | 0.508 |
| **Elderly** | 1.2 [0.7, 1.8] | 0.559 | 1.3 [0.8, 2.3] | 0.261 |
| **In-hospital mortality** |  |  |  |  |
| **Young adults** | Reference |  |  |  |
| **Middle**–**aged adults** | 1.5 [1.0, 2.2] | 0.065 | 1.2 [0.8, 1.9] | 0.023 |
| **Elderly** | 1.6 [1.1, 2.5] | 0.019 | 1.5 [0.9, 2.4] | 0.002 |

Data is presented as odds ratio’s (OR) with a 95 % confidence interval [CI]* Adjusted for: ethnic background, gender, admission type, readmission, direct–hit for ARDS, Charlson Comorbidity Index, APACHE IV score adjusted for age, immunodeficiency, tidal volume per predicted body weight, positive end expiratory pressure. A p–value < 0.05 was considered as statistically significant.

**Table S6** Sequential organ failure scores and information on the restriction on care at the day of death from patients that died on the ICU.

|  | **Young adults**  **(n=44)** | | **Middle–aged adults (n=48)** | | **Elderly**  **(n=46)** | |
| --- | --- | --- | --- | --- | --- | --- |
| SOFA score, median [IQR] | 12 | [5, 16] | 12 | [2, 15] | 13 | [8, 16] |
| SOFA respiratory, median [IQR] | 3 | [1, 4] | 3 | [0, 3] | 3 | [2, 4] |
| SOFA cardiovascular, median [IQR] | 4 | [0, 4] | 4 | [0, 4] | 4 | [3, 4] |
| SOFA nervous system, median [IQR] | 0 | [0, 0] | 0 | [0, 0] | 0 | [0, 4] |
| SOFA liver, median [IQR] | 0 | [0, 1] | 0 | [0, 2] | 0 | [0, 0] |
| SOFA coagulation, median [IQR] | 2 | [0, 3] | 0 | [0, 3] | 1 | [0, 3] |
| SOFA kidney, median [IQR] | 1 | [0, 4] | 2 | [0, 4] | 3 | [1, 4] |
| Restriction on care, n/total n (%) | 26/44 | (59) | 30/48 | (63) | 30/46 | (65) |
| No resuscitation, n/total n (%) | 3/44 | (7) | 3/48 | (6) | 2/46 | (4) |
| Extended restrictions, n/total n (%) | 7/44 | (16) | 10/48 | (21) | 8/46 | (17) |
| Palliative care, n/total n (%) | 16/44 | (36) | 17/48 | (35) | 20/46 | (43) |

SOFA, Sequential organ failure scores; IQR, interquartile range


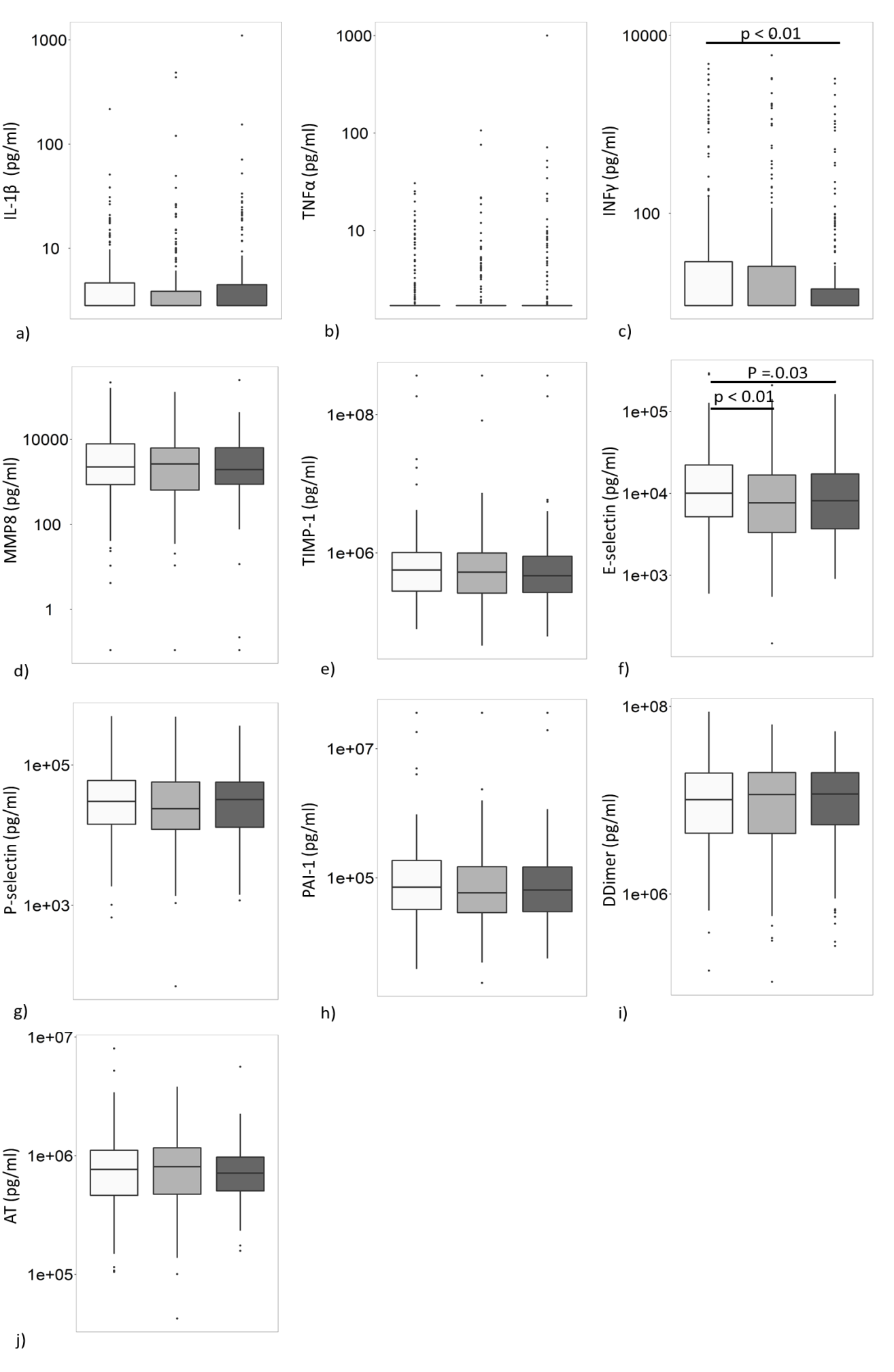
**Figure S2** Biomarker levels stratified by age-groups

**
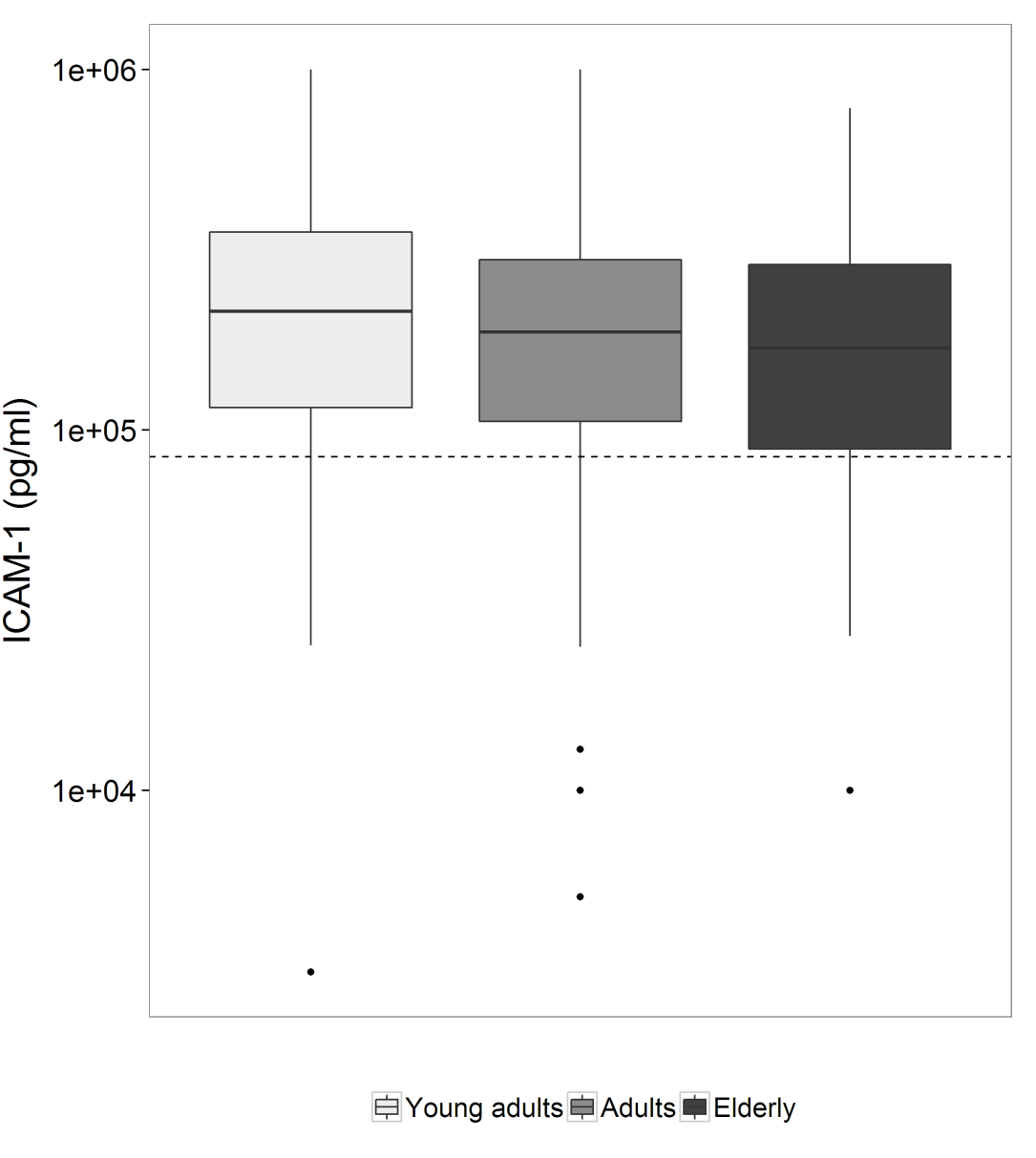

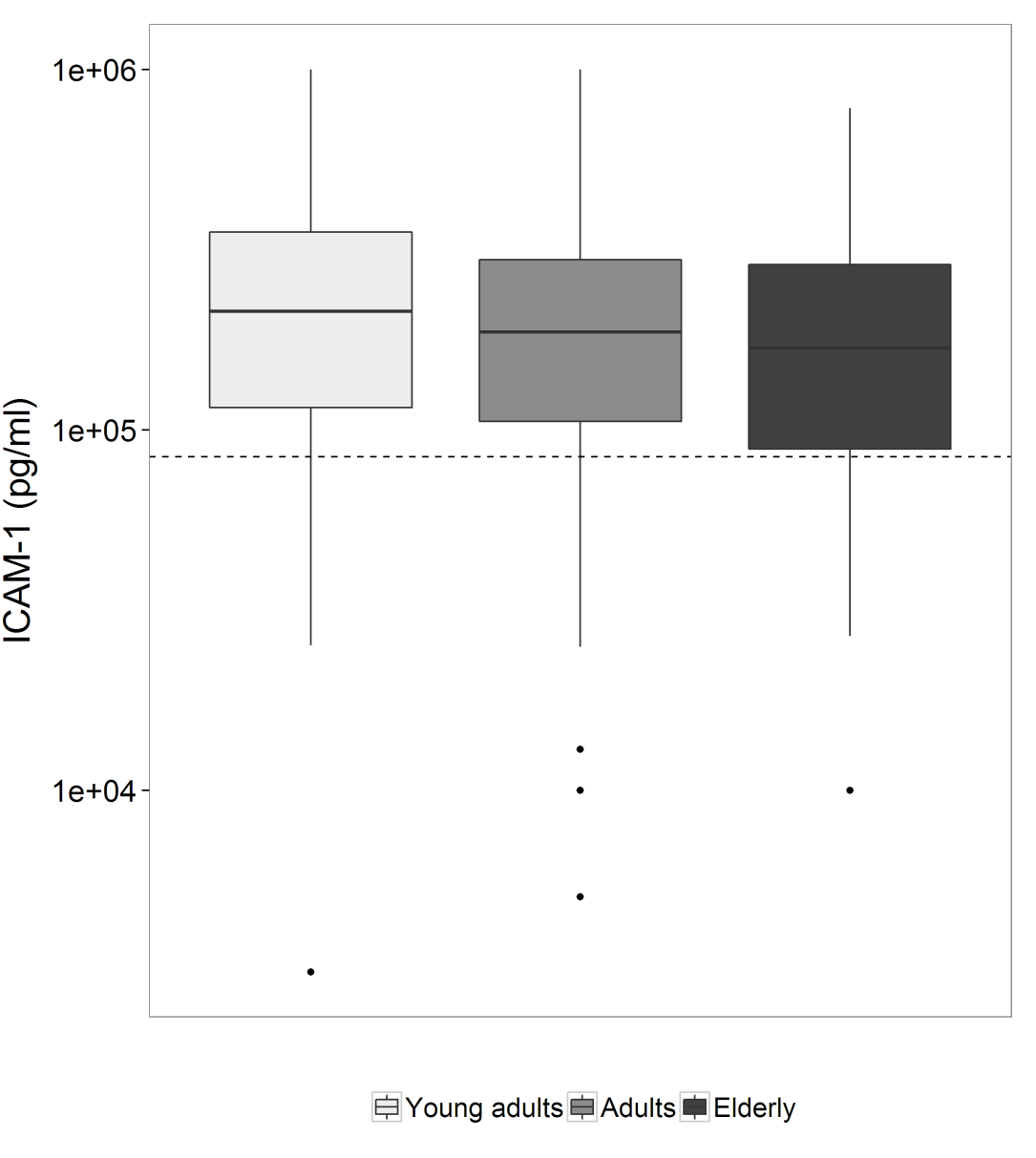

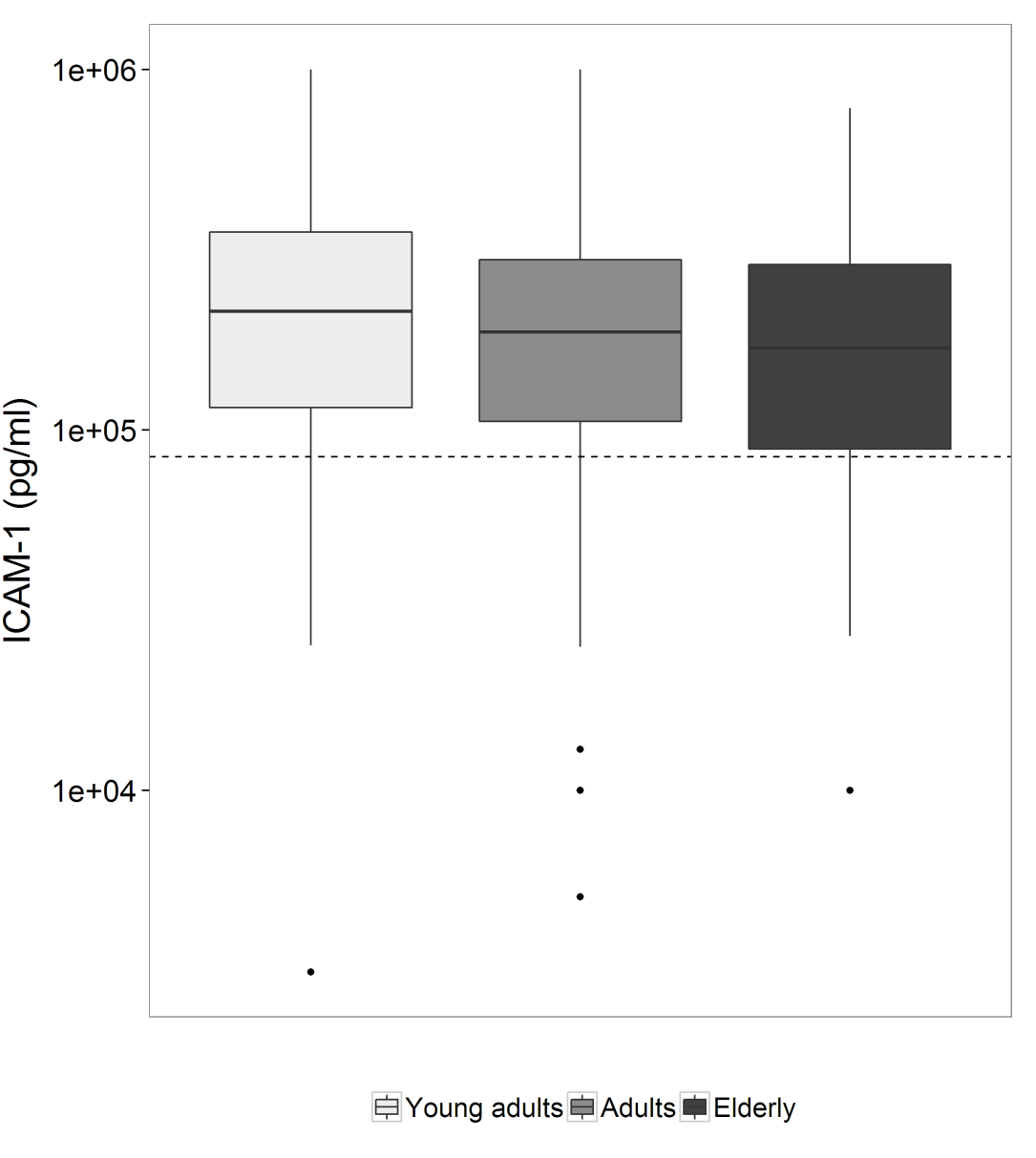
**Systemic levels of a) Interleukin (IL)–1β b) tumor necrosis factor (TNF)–α c) Interferon (INF)–γ, d) matrix metalloproteinase (MMP)–8, e) metallopeptidase inhibitor (TIMP)–1, f) E–selectin, g) P–selectin, h) Plasminogen activator inhibitor (PAI)–1, i) DDimer, j) Anti–thrombin (AT) . Young adults Middle aged adults Elderly. Box and whisker diagrams depict the median and lower quartile, upper quartile, and their respective 1.5 IQR as whiskers – as specified by Tukey. Group differences between young adults and middle aged adults, and young adults and elderly were tested by a Mann–Whitney U test. A p–value < 0.05 was considered as statistically significant.

**Table S7** Association between systemic biomarkers – 90 days mortality in ARDS patients (*Mediation step 2*)

|  | **Crude odds ratio** | |  | **Adjusted odds ratio*** |  |
| --- | --- | --- | --- | --- | --- |
| **Inflammation** | Odds [95% CI] | | P–value | Odds [95% CI] | P–value |
| **IL–6** | 1.14 [1.06, 1.24] | | <0.001 | 1.06 [1.00, 1.17] | 0.156 |
| **IL–8** | 1.32 [1.20, 1.45] | | <0.001 | 1.22 [1.09, 1.36] | <0.001 |
| **IL–10** | 1.29 [1.17, 1.43] | | <0.001 | 1.19 [1.06, 1.33] | 0.003 |
| **IL1–β** | 1.39 [1.12, 1.72] | | 0.003 | 1.24 [1.00, 1.46] | 0.062 |
| **TNFα** | 1.20 [0.94, 1.52] | | 0.139 | 1.12 [0.87, 1.43] | 0.374 |
| **IFNγ** | 1.07 [0.97, 1.20] | | 0.187 | 1.01 [0.91, 1.14] | 0.738 |
| **MMP–8** | 1.03 [0.96, 1.12] | | 0.398 | 1.00 [0.93, 1.10] | 0.812 |
| **TIMP–1** | 1.18 [1.05, 1.33] | | 0.007 | 1.05 [0.92, 1.20] | 0.423 |
| **Endothelial activation markers** |  | |  |  |  |
| **Fractalkine** | 1.58 [1.36, 1.83] | | <0.001 | 1.42 [1.20, 1.67] | <0.001 |
| **E–selectin** | 0.93 [0.80, 1.07] | | 0.313 | 0.89 [0.76, 1.03] | 0.106 |
| **P–selectin** | 0.99 [0.86, 1.14] | | 0.877 | 0.99 [0.84, 1.15] | 0.988 |
| **ICAM–1** | | 1.18 [0.96, 1.46] | 0.120 | 1.01 [0.81, 1.27] | 0.874 |
| **ANG2:ANG1** | | 1.12 [1.05, 1.22] | 0.002 | 1.08 [1.00, 1.16] | 0.046 |
| **Coagulation** | |  |  |  |  |
| **PF4** | | 0.89 [0.83, 0.95] | <0.001 | 0.93 [0.87, 1.00] | 0.069 |
| **DDimer** | | 1.05 [0.90, 1.23] | 0.500 | 1.00 [0.85, 1.17] | 0.980 |
| **tPA** | | 1.58 [1.34, 1.87] | <0.001 | 1.43 [1.20, 1.70] | <0.001 |
| **PAI­–1** | | 1.33 [1.20, 1.47] | <0.001 | 1.27 [1.14, 1.42] | <0.001 |
| **Protein C** | | 1.11 [0.79, 1.57] | 0.552 | 1.20 [0.83, 1.74] | 0.329 |
| **AT** | | 1.03 [0.80, .31] | 0.837 | 1.06 [0.81, 1.38] | 0.681 |

Data is presented as odds ratio’s (OR) with a 95 % confidence interval [CI]. Log transformed biomarkers values. **^.^***Adjusted for: ethnic background, gender, admission type, readmission, direct–hit for ARDS, Charlson Comorbidity Index, APACHE IV score adjusted for age, immunodeficiency, tidal volume per predicted body weight, positive end expiratory pressure. A p–value < 0.05 was considered as statistically significant. IL, interleukin; TNF–α ,tumor necrosis factor alpha; INF–γ, interferon gamma; MMP–8, matrix metalloproteinase–8; TIMP–1, metallopeptidase inhibitor–1; ICAM–1, intracellular adhesion molecule–1; ANG–2:ANG1, angiopoetin–2: angiopoetin–1; PF4, platelet factor-4; tPA, tissue plasminogen activator; PAI–1, Plasminogen activator inhibitor–1; AT, antithrombin. Goodness of fit was tested by omnibus test of fit in multiple regression models, indicating a good fit with p-value 0.716, for all models.

| **Univariate regression model** | | | | | | | | |
| --- | --- | --- | --- | --- | --- | --- | --- | --- |
|  | **Total effect [95% CI]** | **ADE [95% CI]** | **p–value** | **ACME [95% CI]** | **p–value** | **Proportion of mediation [95% CI]** | **p–value** | **p-value, BH correction** |
| **IL–8** |  |  |  |  |  |  |  |  |
| **Middle–aged** | 0.06 [0.03, 0.15] | 0.08 [–0.01, 0.16] | 0.084 | –0.01 [0.04, 0.01] | 0.322 | –0.13 [–2.51, 1.12] | 0.452 | 0.542 |
| **Elderly** | 0.13 [0.04, 0.22] | 0.16 [0.07, 0.25] | <0.001 | –0.03 [–0.06, –0.01] | 0.002 | –0.23 [–1.04, -0.06] | 0.006 | 0.036 |
| **IL-10** |  |  |  |  |  |  |  |  |
| **Middle–aged** | 0.06 [–0.03, 0.15] | 0.08 [–0.01, 0.17] | 0.065 | –0.02 [–0.01, 0.00] | 0.074 | –0.21 [–3.36, 2.56] | 0.230 | 0.328 |
| **Elderly** | 0.13 [0.04, 0.22] | 0.15 [0.06, 0.24] | 0.002 | –0.02[–0.05, –0.01] | 0.016 | –0.19 [–0.95, –0.03] | 0.016 | 0.064 |
| **Fractalkine** |  |  |  |  |  |  |  |  |
| **Middle–aged** | 0.07 [–0.01, 0.16] | 0.10 [0.01, 0.18] | 0.028 | –0.03 [–0.06, –0.00] | 0.014 | –0.32 [–2.38, 3.35] | 0.120 | 0.209 |
| **Elderly** | 0.13 [0.04, 0.22] | 0.18 [0.09, 0.27] | <0.001 | –0.05[–0.08, –0.02] | <0.001 | –0.35 [–1.35, –0.12] | 0.004 | 0.036 |
| **PF4** |  |  |  |  |  |  |  |  |
| **Middle–aged** | 0.07 [-0.01, 0.16] | 0.07 [–0.02, 0.16] | 0.106 | –0.01 [–0.02, 0.01] | 0.394 | –0.07 [–0.45, 1.27] | 0.521 | 0.568 |
| **Elderly** | 0.13 [0.04, 0.21] | 0.14 [0.05, 0.23] | 0.002 | –0.02 [–0.04, –0.02] | 0.030 | –0.12 [–0.57, –0.01] | 0.042 | 0.126 |
| **PAI-1** |  |  |  |  |  |  |  |  |
| **Middle–aged** | 0.07 [–0.02, 0.16] | 0.09 [–0.00, 0.17] | 0.064 | 0.02 [–0.05, 0.00] | 0.081 | –0.24 [–3.22, 2.13] | 0.246 | 0.328 |
| **Elderly** | 0.15[0.06, 0.23] | 0.15 [0.06, 0.23] | 0.002 | -0.02 [–0.05, 0.00] | 0.068 | –0.17 [–0.78, 0.02] | 0.072 | 0.173 |
| **tPA** |  |  |  |  |  |  |  |  |
| **Middle–aged** | 0.06 [–0.02, 0.16] | 0.06 [–0.03, 0.15] | 0.214 | 0.00[–0.02, 0.03] | 0.673 | 0.06 [–0.38, 1.16] | 0.686 | 0.686 |
| **Elderly** | 0.13 [0.04, 0.23] | 0.11 [0.02, 0.20] | 0.017 | 0.02 [–0.00, 0.04] | 0.116 | 0.13 [–0.03, 0.45] | 0.122 | 0.209 |

**Table S8**. Mediation pathway analysis – age, systemic biomarkers and mortality, univariate *(Mediation step 4)*

Data is presented as estimate with a 95 % confidence interval [CI]. ADE, average direct effect; ACME, average causal mediation effect. A p–value < 0.05 was considered as statistical significant. There was no significant exposure–mediator interaction for any mediator. BH, Benjamini-Hochberg; IL–8, Interleukin–8; IL–10, Interleukin–10; PF4, platelet factors 4; PAI, Plasminogen activator inhibitor–1; tPA, tissue plasminogen activator.

**Table S9**. Mediation pathway analysis – age, systemic biomarkers and mortality, multiple regression models *(Mediation step 4)*

| **Multiple regression models^‡^** | | | | | | | | |
| --- | --- | --- | --- | --- | --- | --- | --- | --- |
|  | **Total effect [95% CI]** | **ADE [95% CI]** | **p–value** | **ACME [95% CI]** | **p–value** | **Proportion of mediation [95% CI]** | **p-value** | **p-value, BH correction** |
| **IL–8** |  |  |  |  |  |  |  |  |
| **Middle–aged** | 0.08 [0.01, 0.17] | 0.08 [0.00, 0.17] | 0.056 | -0.00 [-0.02, 0.01] | 0.640 | –0.03 [–0.61, 0.23] | 0.625 | 0.625 |
| **Elderly** | 0.16 [0.07, 0.26] | 0.17 [0.08, 0.27] | <0.001 | -0.01 [-0.03, 0.00] | 0.130 | –0.05 [–0.23, 0.02] | 0.120 | 0.325 |
| **IL–10** |  |  |  |  |  |  |  |  |
| **Middle–aged** | 0.09 [0.01, 0.17] | 0.09 [–0.00, 0.18] | 0.063 | -0.00 [-0.02, 0.00] | 0.150 | –0.08 [–0.65, 0.88] | 0.224 | 0.325 |
| **Elderly** | 0.16 [0.07, 0.26] | 0.17 [0.07, 0.26] | 0.004 | -0.01 [-0.03, 0.00] | 0.098 | –0.05 [–0.24, 0.00] | 0.072 | 0.288 |
| **Fractalkine** |  |  |  |  |  |  |  |  |
| **Middle–aged** | 0.09 [0.00, 0.18] | 0.10 [0.02, 0.19] | 0.026 | -0.01 [-0.03, 0.00] | 0.089 | –0.10 [–1.16, –0.32] | 0.204 | 0.325 |
| **Elderly** | 0.17 [0.10, 0.26] | 0.19 [0.10, 0.27] | <0.001 | –0.02 [-0.04, -0.00] | 0.020 | –0.11 [–0.37, –0.01] | 0.020 | 0.120 |
| **PF4** |  |  |  |  |  |  |  |  |
| **Middle–aged** | 0.08 [–0.00, 0.17] | 0.09 [0.00, 0.17] | 0.056 | –0.00[–0.01, 0.00] | 0.392 | –0.03 [–0.59, 0.28] | 0.400 | 0.480 |
| **Elderly** | 0.16[0.07, 0.25] | 0.16 [0.07, 0.25] | 0.006 | –0.00[–0.02, 0.00] | 0.273 | –0.03 [–0.17, 0.02] | 0.208 | 0.325 |
| **PAI–1** |  |  |  |  |  |  |  |  |
| **Middle–aged** | 0.08 [0.00, 0.17] | 0.09 [0.01, 0.17] | 0.030 | –0.01[–0.03, 0.01] | 0.215 | –0.12 [–1.15, 0.28] | 0.240 | 0.325 |
| **Elderly** | 0.16 [0.07, 0.25] | 0.17 [0.07, 0.26] | <0.001 | –0.01[–0.03, 0.01] | 0.258 | –0.06 [–0.32, 0.04] | 0.244 | 0.325 |
| **tPA** |  |  |  |  |  |  |  |  |
| **Middle–aged** | 0.09 [0.00, 0.18] | 0.09 [–0.01, 0.16] | 0.082 | 0.00 [–0.01, 0.02] | 0.557 | 0.04 [–0.22, 0.42] | 0.492 | 0.537 |
| **Elderly** | 0.16 [0.06, 0.25] | 0.14 [0.05, 0.23] | 0.008 | 0.02 [0.00, 0.03] | 0.016 | 0.10 [0.01, 0.28] | 0.018 | 0.120 |

Data is presented as estimate with a 95 % confidence interval [CI]. ADE, average direct effect; ACME, average causal mediation effect. Adjusted for: ethnic background, gender, admission type, readmission, direct–hit for ARDS, Charlson Comorbidity Index, APACHE IV score adjusted for age, immunodeficiency, tidal volume per predicted body weight, positive end expiratory pressure. A p–value < 0.05 was considered as statistical significant. There was no significant exposure–mediator interaction for any mediator. BH, Benjamini-Hochberg; IL–8, Interleukin–8; IL–10, Interleukin–10; PF4, platelet factor 4; PAI–1, Plasminogen activator inhibitor–1; tPA, tissue plasminogen activator.

**Sensitivity analysis:**

***Subgroup analysis of patients with pulmonary ARDS:***

**Table S10** Covariates of patients with pulmonary ARDS

|  | **Young adults**  **(n=142)** | | **Middle–age adults**  **(n=137**) | | **Elderly**  **(n=125**) | | **p–value** |
| --- | --- | --- | --- | --- | --- | --- | --- |
| **Demographics** |  |  |  |  |  |  |  |
| Age (years), Median [IQR] | 43 | [32, 50] | 61 | [58, 64] | 74 | [71, 78] | <0.01 |
| Male, n/total n (%) | 83 | (58) | 93 | (68) | 87 | (70) | 0.10 |
| Race, Caucasian, n/total n (%) | 117 | (82) | 120 | (88) | 121 | (97) | <0.01 |
| Admission type, Medical, n/total n (%) | 120 | (85) | 110 | (80) | 93 | (74) | 0.13 |
| Readmission, n/total n (%) | 15 | (11) | 17 | (12) | 24 | (19) | 0.11 |
| Charlson Comorbidity Index, Median [IQR] | 0 | [0, 2] | 1 | [0, 2] | 0 | [0, 2] | 0.30 |
| APACHE IV Score adjusted for age, Median [IQR] | 74 | [56, 100] | 71 | [51, 96] | 67 | [51, 86] | 0.27 |
| Tidal volume per PBW, Median [IQR] | 7.1 | [6.2, 8.2] | 7.0 | [6.2, 8.4] | 7.0 | [6.4, 8.0] | 0.82 |
| PEEP (cmH_2_O), Median [IQR] | 10 | [8, 14] | 10 | [8, 12] | 9 | [7, 12] | 0.10 |
| PaO_2_ FiO_2_ ratio, Median [IQR] | 160 | [120, 205] | 160 | [107, 217] | 157 | [116, 211] | 0.98 |

IQR, interquartile range; APACHE, acute physiology and chronic health evaluation; PBW, predicted body weight; PEEP, positive end-expiratory pressure; A p–value < 0.05 was considered as statistically significant.

**Table S11** Association between age and mortality in patients with pulmonary ARDS (*Mediation step 1*)

|  | **Crude odds ratio [95% CI]** | **p–value** | **Adjusted odds ratio [95% CI]** ^‡^ | **p–value** |
| --- | --- | --- | --- | --- |
| **90 days mortality** |  |  |  |  |
| **Young adults** | Reference | NA | Reference | NA |
| **Middle**–**aged adults** | 1.6 [0.9, 2.6] | 0.08 | 1.7 [1.0, 2.8] | 0. 07 |
| **Elderly** | 1.9 [1.1, 3.1] | 0.02 | 2.3 [1.3, 4.1] | <0.01 |
| Data is presented as odds ratio’s (OR) with a 95 % confidence interval [CI]. Number of patients per age group: young adults n=142, middle-aged adults n=137 and elderly n=125. **^‡^** Adjusted for: ethnic background, gender, admission type, readmission, Charlson Comorbidity Index, APACHE IV score adjusted for age, immunodeficiency, tidal volume per predicted body weight, positive end expiratory pressure. A p–value < 0.05 was considered as statistically significant.  NA, not applicable. | | | | |

**Table S12** Association between systemic biomarkers – 90 days mortality in patients with pulmonary ARDS (*Mediation step 2*)

|  | **Crude odds ratio** | |  | **Adjusted odds ratio**^‡^ |  |
| --- | --- | --- | --- | --- | --- |
| **Inflammation** | Odds [95%] | | P–value | Odds [95%] | P–value |
| **IL–6** | 1.15 [1.04, 1.27] | | <0.01 | 1.11 [0.99, 1.25] | 0.05 |
| **IL–8** | 1.33 [1.19, 1.50] | | <0.01 | 1.29 [1.13, 1.49] | <0.01 |
| **IL–10** | 1.32 [1.16, 1.51] | | <0.01 | 1.28 [1.10, 1.49] | <0.01 |
| **IL1–β** | 1.68 [1.23, 2.30] | | <0.01 | 1.56 [1.12, 2.19] | 0.01 |
| **TNFα** | 1.29 [0.93, 1.77] | | 0.12 | 1.22 [0.87, 1.71] | 0.24 |
| **IFNγ** | 1.04 [0.91, 1.19] | | 0.55 | 1.00 [0.86, 1.15] | 0.92 |
| **MMP–8** | 1.05 [0.95, 1.16] | | 0.34 | 1.04 [0.94, 1.15] | 0.47 |
| **TIMP–1** | 1.21 [1.03, 1.42] | | 0.02 | 1.09 [0.92, 1.30] | 0.27 |
| **Endothelial activation markers** |  | |  |  |  |
| **Fractalkine** | 1.61 [1.35, 1.92] | | <0.01 | 1.49 [1.23, 1.81] | <0.01 |
| **E–selectin** | 0.91 [0.75, 1.11] | | 0.35 | 0.91 [0.74, 1.12] | 0.33 |
| **P–selectin** | 0.98 [0.81, 1.17] | | 0.79 | 0.98 [0.81, 1.20] | 0.79 |
| **ICAM–1** | | 1.26 [0.96, 1.66] | 0.10 | 1.12 [0.83, 1.52] | 0.34 |
| **ANG2:ANG1** | | 1.13 [1.03, 1.24] | <0.01 | 1.09 [1.00, 1.20] | 0.05 |
| **Coagulation** | |  |  |  |  |
| **PF4** | | 0.86 [0.80, 0.94] | <0.01 | 0.90 [0.82, 0.98] | 0.01 |
| **DDimer** | | 0.98 [0.82, 1.18] | 0.86 | 0.96 [0.78, 1.17] | 0.73 |
| **tPA** | | 1.51 [1.23, 1.84] | <0.01 | 1.36 [1.11, 1.67] | <0.01 |
| **PAI­–1** | | 1.29 [1.14, 1.45] | <0.01 | 1.25 [1.09, 1.43] | <0.01 |
| **Protein C** | | 1.14 [0.76, 1.71] | 0.53 | 1.21 [0.79, 1.88] | 0.38 |
| **AT** | | 1.01 [0.73, 1.40] | 0.94 | 1.03 [0.74, 1.46] | 0.78 |
| Data is presented as odds ratio’s (OR) with a 95 % confidence interval [CI]. Log transformed biomarkers values. Number of patients per age group: young adults n=142, middle-aged adults n=137 and elderly n=125. ^‡^Adjusted for: ethnic background, gender, admission type, readmission, Charlson Comorbidity Index, APACHE IV score adjusted for age, immunodeficiency, tidal volume per predicted body weight, positive end expiratory pressure. A p–value < 0.05 was considered as statistical significant. IL, interleukin; TNF–α, tumor necrosis factor alpha; INF–γ, interferon gamma; MMP–8, matrix metalloproteinase–8; TIMP–1, metallopeptidase inhibitor–1; ICAM–1, intracellular adhesion molecule–1; ANG–2:ANG1, angiopoetin–2: angiopoetin–1; PF4, platelet factor–4; tPA, tissue plasminogen activator; PAI–1, Plasminogen activator inhibitor–1; AT, antithrombin | | | | | |

**Table S13** Association between age and biomarker levels of patients with pulmonary ARDS *(Mediation Step 3)*

|  | **Coefficient [95% CI]** | **p–value** | **Coefficient [95% CI]** ^‡^ | **p–value** |
| --- | --- | --- | --- | --- |
| **IL–6** |  |  |  |  |
| **Young adults** | Reference | NA | Reference | NA |
| **Middle–aged adults** | –0.34 [–0.82, 0.14] | 0.17 | –0.31 [–0.76, 0.13] | 0.13 |
| **Elderly** | –0.33 [–0.83, 0.16] | 0.18 | –0.31 [–0.78, 0.16] | 0.17 |
| **IL–8** |  |  |  |  |
| **Young adults** | Reference | NA | Reference | NA |
| **Middle–aged adults** | –012 [–0.55, 0.31] | 0.59 | –0.03 [–0.42, 0.35] | 0.86 |
| **Elderly** | –0.40 [–0.84, 0.04] | 0.08 | –0.23 [–0.64, 0.17] | 0.31 |
| **IL–10** |  |  |  |  |
| **Young adults** | Reference | NA | Reference | NA |
| **Middle–aged adults** | –0.17 [0.55, 0.20] | 0.37 | –0.09 [–0.43, 0.25] | 0.60 |
| **Elderly** | –0.14 [0.53, 0.24] | 0.47 | –0.01 [–0.37, 0.35] | 0.92 |
| **IL–1β** |  |  |  |  |
| **Young adults** | Reference | NA | Reference | NA |
| **Middle–aged adults** | –0.01 [–0.20, 0.16] | 0.98 | –0.03 [–0.13, 0.18] | 0.71 |
| **Elderly** | –0.02 [–0.14, 0.19] | 0.78 | 0.07 [–0.07, 0.26] | 0.19 |
| **TNFα** |  |  |  |  |
| **Young adults** | Reference | NA | Reference | NA |
| **Middle–aged adults** | –0.01 [–0.15, 0.13] | 0.87 | 0.01 [–0.14, 0.15] | 0.89 |
| **Elderly** | –0.10 [–0.05, 0.25] | 0.18 | 0.15 [–0.01, 0.30] | 0.06 |
| **INFγ** |  |  |  |  |
| **Young adults** | Reference | NA | Reference | NA |
| **Middle–aged adults** | –0.28 [–0.63, 0.07] | 0.12 | –0.21 [–0.56, 0.14] | 0.23 |
| **Elderly** | –0.52 [–0.87, –0.16] | <0.01 | –0.36 [–0.73, 0.00] | 0.05 |
| **MMP–8** |  |  |  |  |
| **Young adults** | Reference | NA | Reference | NA |
| **Middle–aged adults** | –0.13 [–0.63, 0.37] | 0.62 | –0.07 [–0.56, 0.43] | 0.85 |
| **Elderly** | –0.06 [–0.46, 0.57] | 0.83 | 0.17 [–0.35, 0.69] | 0.49 |
| **TIMP–1** |  |  |  |  |
| **Young adults** | Reference | NA | Reference | NA |
| **Middle–aged adults** | –0.06 [–0.37, 0.24] | 0.70 | –0.02 [–0.30, 0.27] | 0.96 |
| **Elderly** | –0.13 [–0.44, 0.18] | 0.41 | 0.01 [–0.30–0.31] | 0.84 |
| **Fractalkine** |  |  |  |  |
| **Young adults** | Reference | NA | Reference | NA |
| **Middle–aged adults** | –0.23 [–0.52, 0.06] | 0.12 | –0.16 [–0.44, 0.10] | 0.25 |
| **Elderly** | –0.44 [–0.69, –0.15] | <0.01 | –0.26 [–0.55, 0.02] | 0.07 |
| **E–selectin** |  |  |  |  |
| **Young adults** | Reference | NA | Reference | NA |
| **Middle–aged adults** | –0.17 [–0.42, 0.08] | 0.18 | –0.27 [–0.39, 0.10] | 0.24 |
| **Elderly** | –0.14 [–0.39, 0.12] | 0.29 | –0.09 [–0.35, 0.17] | 0.49 |
| **P–selectin** |  |  |  |  |
| **Young adults** | Reference | NA | Reference | NA |
| **Middle–aged adults** | –0.10 [–0.36, 0.16] | 0.43 | –0.05 [–0.33, 0.18] | 0.70 |
| **Elderly** | 0.19 [–0.07, 0.46] | 0.16 | 0.20 [–0.07, 0.47] | 0.16 |
| **ICAM–1** |  |  |  |  |
| **Young adults** | Reference | NA | Reference | NA |
| **Middle–aged adults** | –0.08 [–0.26, 0.09] | 0.36 | –0.04 [–0.22, 0.13] | 0.62 |
| **Elderly** | –0.22 [–0.40, –0.04] | 0.01 | –0.14 [–0.33, 0.03] | 0.11 |
| **Ang2:Ang1** |  |  |  |  |
| **Young adults** | Reference | NA | Reference | NA |
| **Middle–aged adults** | –0.19 [–0.75, 0.37] | 0.51 | –0.22 [–0.78, 0.35] | 0.49 |
| **Elderly** | –0.12 [–0.70, 0.45] | 0.68 | –0.08 [–0.68, 0.51] | 0.80 |
| **Platelet factor 4** |  |  |  |  |
| **Young adults** | Reference | NA | Reference | NA |
| **Middle–aged adults** | 0.42 [–0.22, 1.06] | 0.20 | 0.47 [–0.12, 1.07] | 0.12 |
| **Elderly** | 0.71 [0.06, 1.37] | 0.03 | 0.62 [–0.01, 1.25] | 0.06 |
| **D-Dimer** |  |  |  |  |
| **Young adults** | Reference | NA | Reference | NA |
| **Middle–aged adults** | 0.09 [-0.18, 0.34] | 0.52 | 0.10 [-0.15, 0.36] | 0.44 |
| **Elderly** | 0.15 [-0.12, 0.41] | 0.28 | 0.17 [-0.11, 0.24] | 0.28 |
| **Tissue plasminogen activator** |  |  |  |  |
| **Young adults** | Reference | NA | Reference | NA |
| **Middle–aged adults** | 0.01 [-0.24, 0.27] | 0.91 | 0.01 [-0.25, 0.27] | 0.94 |
| **Elderly** | -0.10 [-0.16, 0.37] | 0.45 | 0.16 [-0.11, 0.43] | 0.24 |
| **PAI–1** |  |  |  |  |
| **Young adults** | Reference | NA | Reference | NA |
| **Middle–aged adults** | -0.17 [-0.57, 0.24] | 0.42 | -0.11 [-0.49, 0.28] | 0.67 |
| **Elderly** | -0.26 [-0.67, 0.16] | 0.23 | -0.19 [-0.60, 0.21] | 0.43 |
| **Protein C** |  |  |  |  |
| **Young adults** | Reference | NA | Reference | NA |
| **Middle–aged adults** | -0.04 [-0.07, 0.16] | 0.46 | 0.04 [-0.08, 0.16] | 0.51 |
| **Elderly** | -0.00 [-0.13, 0.12] | 0.94 | -0.03 [-0.16, 0.09] | 0.58 |
| **Anti–thrombin** |  |  |  |  |
| **Young adults** | Reference | NA | Reference | NA |
| **Middle–aged adults** | -0.03 [-0.17, 0.12] | 0.74 | -0.03 [-0.18, 0.12] | 0.62 |
| **Elderly** | -0.08 [-0.22, 0.08] | 0.33 | -0.08 [-0.23, 0.08] | 0.32 |
| Data is presented as beta-coefficient (β) with a 95 % confidence interval [CI]. All biomarkers are log–transformed. Number of patients per age group: young adults n=142, middle-aged adults n=137 and elderly n=125. ‡Adjusted for: ethnic background, gender, admission type, readmission, Charlson Comorbidity Index, APACHE-IV score adjusted for age, immunodeficiency, tidal volume per predicted body weight, positive end expiratory pressure. A p–value < 0.05 was considered as statistical significant. IL, interleukin; TNF–α ,tumor necrosis factor alpha; INF–γ, interferon gamma; MMP–8, matrix metalloproteinase–8, TIMP–1, metallopeptidase inhibitor–1, ICAM–1, intracellular adhesion molecule–1; ANG–2:ANG1, angiopoetin–2: angiopoetin–1, PAI–1, Plasminogen activator inhibitor–1. NA, not applicable. | | | | |

**Table S14**. Mediation pathway analysis – age, systemic biomarkers and mortality, univariate and multiple regression models of patients with pulmonary ARDS *(Mediation step 4)*

|  |  | |  | |  | | **Univariate regression** | | |  |  | |  |  |
| --- | --- | --- | --- | --- | --- | --- | --- | --- | --- | --- | --- | --- | --- | --- |
|  | **Total effect [95% CI]** | **ADE [95% CI]** | | **p–value** | | **ACME [95% CI]** | | **p–value** | **Proportion of mediation**  **[95% CI]** | | | **p-value** | | **p-value, BH correction** |
| **Fractalkine** |  |  | |  | |  | |  |  | | |  | |  |
| **Middle–aged** | 0.11 [-0.01, 0.22] | 0.13 [0.02, 0.24] | | 0.014 | | –0.02 [–0.06, 0.01] | | 0.152 | –0.22 [–2.92, 0.82] | | | 0.222 | | 0.296 |
| **Elderly** | 0.15 [0.03, 0.26] | 0.19 [0.09, 0.30] | | 0.002 | | –0.05[–0.08, –0.02] | | 0.004 | –0.31 [1.53, –0.08] | | | 0.014 | | 0.056 |
| **PF4** |  |  | |  | |  | |  |  | | |  | |  |
| **Middle–aged** | 0.10 [0.01, 0.21] | 0.11 [0.01, 0.22] | | 0.040 | | –0.01 [–0.04, 0.01] | | 0.254 | –0.12 [–1.60, 0.58] | | | 0.328 | | 0.328 |
| **Elderly** | 0.14 [0.02, 0.25] | 0.17 [0.04, 0.28] | | 0.006 | | –0.02 [–0.05, 0.00] | | 0.032 | –0.16 [–0.78, 0.01] | | | 0.046 | | 0.092 |

| **Multiple regression‡** | | | | | | | | |
| --- | --- | --- | --- | --- | --- | --- | --- | --- |
|  | **Total effect [95% CI]** | **ADE [95% CI]** | **p–value** | **ACME [95% CI]** | **p–value** | **Proportion of mediation**  **[95% CI]** | **p-value** | **p-value, BH correction** |
| **Fractalkine** |  |  |  |  |  |  |  |  |
| **Middle–aged** | 0.10 [0.00, 0.20] | 0.11 [0.01, 0.22] | 0.043 | –0.01 [–0.04, 0.01] | 0.212 | –0.13 [–1.28, 0.48] | 0.284 | 0.284 |
| **Elderly** | 0. 18 [0.06, 0.29] | 0.20 [0.09, 0.31] | <0.001 | –0.02 [–0.05, 0.00] | 0.064 | –0.14 [–0.56, 0.00] | 0.064 | 0.156 |
| **PF4** |  |  |  |  |  |  |  |  |
| **Middle–aged** | 0.10 [0.01, 0.20] | 0.11 [0.01, 0.21] | 0.030 | –0.01 [–0.03, 0.00] | 0.123 | –0.10 [–1.07, 0.42] | 0.230 | 0.284 |
| **Elderly** | 0.17 [0.06, 0.28] | 0.19 [0.06, 0.28] | <0.001 | –0.02[–0.04, 0.00] | 0.076 | –0.09 [–0.38, 0.00] | 0.078 | 0.156 |

Data is presented as estimate with a 95 % confidence interval [CI]. Number of patients per age group: young adults n=142, middle-aged adults n=137 and elderly n=125. ADE, average direct effect; ACME, average causal mediation effect. Adjusted for: ethnic background, gender, admission type, readmission, Charlson Comorbidity Index, APACHE IV score adjusted for age, immunodeficiency, tidal volume per predicted body weight, positive end expiratory pressure. A p–value < 0.05 was considered as statistically significant. There was no significant exposure–mediator interaction for any mediator. BH, Benjamini-Hochberg, IL–8, Interleukin–8; IL–10, Interleukin–10; PF4, platelet factor 4; PAI–1, Plasminogen activator inhibitor–1; tPA, tissue plasminogen activator.

***Adjusted analysis without CCI and comorbidity as covariates***

**Table S15** Association between age and mortality without CCI and comorbidity as covariates (*Mediation step 1*)

|  | **Adjusted odds ratio [95% CI]*** | **p–value** |
| --- | --- | --- |
| **90 days mortality** |  |  |
| **Young adults** | reference | NA |
| **Middle**–**aged adults** | 1.5 [1.0, 2.3] | 0. 05 |
| **Elderly** | 2.1 [1.3, 3.2] | <0.01 |
| Data is presented as odds ratio’s (OR) with a 95 % confidence interval [CI]. ‡ Adjusted for: ethnic background, gender, admission type, readmission, direct–hit for ARDS, immunodeficiency, tidal volume per predicted body weight, positive end expiratory pressure. A p–value < 0.05 was considered as statistically significant. NA = not applicable | | |

**Table S16** Association between systemic biomarkers – 90 days mortality in ARDS patients without CCI and comorbidity as covariates (*Mediation step 2*)

|  | **Adjusted odds ratio*** | |  | |  |
| --- | --- | --- | --- | --- | --- |
| **Inflammation** | Odds [95%] | | P–value | |  |
| **IL–6** | 1.13 [1.05, 1.23] | | <0.01 | |  |
| **IL–8** | 1.32 [1.19, 1.46] | | <0.01 | |  |
| **IL–10** | 1.29 [1.16, 1.43] | | <0.01 | |  |
| **IL1–β** | 1.33 [1.07, 1.66] | | <0.01 | |  |
| **TNFα** | 1.16 [0.91, 1.48] | | 0.22 | |  |
| **IFNγ** | 1.05 [0.94, 1.17] | | 0.86 | |  |
| **MMP–8** | 1.03 [0.95, 1.12] | | 0.38 | |  |
| **TIMP–1** | 1.15 [1.01, 1.30] | | 0.03 | |  |
| **Endothelial activation markers** |  | |  | |  |
| **Fractalkine** | 1.57 [1.35, 1.84] | | <0.01 | |  |
| **E–selectin** | 0.91 [0.78, 1.06] | | 0.16 | |  |
| **P–selectin** | 1.01[0.87, 1.17] | | 0.96 | |  |
| **ICAM–1** | | 111 [0.89, 1.38] | | 0.32 | |
| **ANG2:ANG1** | | 1.13 [1.04, 1.21] | | <0.01 | |
| **Coagulation** | |  | |  | |
| **PF4** | | 0.89 [0.83, 0.95] | | <0.01 | |
| **DDimer** | | 1.03 [0.88, 1.21] | | 0.66 | |
| **tPA** | | 1.56 [1.31, 1.84] | | <0.01 | |
| **PAI­–1** | | 1.35 [1.21, 1.50] | | <0.01 | |
| **Protein C** | | 1.17[0.82, 1.67] | | 0.39 | |
| **AT** | | 1.01 [0.78, 1.31] | | 0.92 | |
| Data is presented as odds ratio’s (OR) with a 95 % confidence interval [CI]. Log transformed biomarkers values. **^.^***Adjusted for: ethnic background, gender, admission type, readmission, direct–hit for ARDS, immunodeficiency, tidal volume per predicted body weight, positive end expiratory pressure. A p–value < 0.05 was considered as statistically significant. IL, interleukin; TNF–α ,tumor necrosis factor alpha; INF–γ, interferon gamma; MMP–8, matrix metalloproteinase–8; TIMP–1, metallopeptidase inhibitor–1; ICAM–1, intracellular adhesion molecule–1; ANG–2:ANG1, angiopoetin–2: angiopoetin–1; PF4, platelet factor-4; tPA, tissue plasminogen activator; PAI–1, Plasminogen activator inhibitor–1; AT, antithrombin | | | | | |

**Table S17** Association between age and biomarker levels in ARDS patients without CCI and comorbidity as covariates *(Mediation Step 3)*

|  | **Coefficient [95% CI]*** | **p–value** |
| --- | --- | --- |
| **IL–6** |  |  |
| **Young adults** | Reference | NA |
| **Middle–aged adults** | –0.32 [–0.71, 0.07] | 0.09 |
| **Elderly** | –0.31 [–0.72, 0.10] | 0.18 |
| **IL–8** |  |  |
| **Young adults** | Reference | NA |
| **Middle–aged adults** | –0.05 [–0.39, 0.29] | 0.79 |
| **Elderly** | –0.22 [–0.58, 0.14] | 0.25 |
| **IL–10** |  |  |
| **Young adults** | Reference | NA |
| **Middle–aged adults** | –0.22 [–0.54, 0.09] | 0.17 |
| **Elderly** | –0.26 [–0.59, 0.07] | 0.18 |
| **IL–1β** |  |  |
| **Young adults** | Reference | NA |
| **Middle–aged adults** | –0.02 [–0.17, 0.13] | 0.80 |
| **Elderly** | 0.06 [–0.09, 0.22] | 0.45 |
| **TNFα** |  |  |
| **Young adults** | Reference | NA |
| **Middle–aged adults** | -0.02 [–0.15, 0.11] | 0.72 |
| **Elderly** | 0.08 [–0.05, 0.22] | 0.26 |
| **INFγ** |  |  |
| **Young adults** | Reference | NA |
| **Middle–aged adults** | –0.11 [–0.40, 0.18] | 0.49 |
| **Elderly** | –0.24 [–0.54, 0.06] | 0.10 |
| **MMP–8** |  |  |
| **Young adults** | Reference | NA |
| **Middle–aged adults** | –0.19 [–0.59, 0.21] | 0.36 |
| **Elderly** | 0.17 [–0.35, 0.69] | 0.96 |
| **TIMP–1** |  |  |
| **Young adults** | Reference | NA |
| **Middle–aged adults** | –0.01 [–0.27, 0.24] | 0.79 |
| **Elderly** | 0.02 [–0.25–0.29] | 0.89 |
| **Fractalkine** |  |  |
| **Young adults** | Reference | NA |
| **Middle–aged adults** | –0.18 [–0.40, 0.04] | 0.13 |
| **Elderly** | –0.26 [–0.50, -0.04] | 0.03 |
| **E–selectin** |  |  |
| **Young adults** | Reference | NA |
| **Middle–aged adults** | –0.26 [–0.48, -0.05] | 0.02 |
| **Elderly** | –0.21 [–0.44, 0.02] | 0.09 |
| **P–selectin** |  |  |
| **Young adults** | Reference | NA |
| **Middle–aged adults** | –0.11 [–0.33, 0.11] | 0.39 |
| **Elderly** | 0.03 [–0.19, 0.26] | 0.70 |
| **ICAM–1** |  |  |
| **Young adults** | Reference | NA |
| **Middle–aged adults** | –0.04 [–0.19, 0.11] | 0.51 |
| **Elderly** | –0.13 [–0.28, 0.03] | 0.11 |
| **Ang2:Ang1** |  |  |
| **Young adults** | Reference | NA |
| **Middle–aged adults** | –0.26 [–0.72, 0.20] | 0.28 |
| **Elderly** | –0.17 [–0.65, 0.32] | 0.50 |
| **Platelet factor 4** |  |  |
| **Young adults** | Reference | NA |
| **Middle–aged adults** | 0.23 [–0.27, 0.73] | 0.32 |
| **Elderly** | 0.35 [–0.17, 0.87] | 0.14 |
| **D-Dimer** |  |  |
| **Young adults** | Reference | NA |
| **Middle–aged adults** | -0.01 [-0.22, 0.20] | 0.93 |
| **Elderly** | 0.03 [-0.119, 0.25] | 0.73 |
| **Tissue plasminogen activator** |  |  |
| **Young adults** | Reference | NA |
| **Middle–aged adults** | 0.09 [-0.12, 0.30] | 0.43 |
| **Elderly** | 0.25 [0.03, 0.47] | 0.03 |
| **PAI–1** |  |  |
| **Young adults** | Reference | NA |
| **Middle–aged adults** | -0.21 [-0.55, 0.11] | 0.15 |
| **Elderly** | -0.19 [-0.54, 0.15] | 0.29 |
| **Protein C** |  |  |
| **Young adults** | Reference | NA |
| **Middle–aged adults** | 0.040[-0.09, 0.09] | 0.99 |
| **Elderly** | -0.07 [-0.17, 0.03] | 0.13 |
| **Anti–thrombin** |  |  |
| **Young adults** | Reference | NA |
| **Middle–aged adults** | 0.01 [-0.11, 0.14] | 0.96 |
| **Elderly** | -0.03 [-0.16, 0.10] | 0.52 |
| Data is presented as beta-coefficient (β) with a 95 % confidence interval [CI]. All biomarkers are log–transformed. ‡Adjusted for: ethnic background, gender, admission type, readmission, direct-hit for ARDS, immunodeficiency, tidal volume per predicted body weight, positive end expiratory pressure. A p–value < 0.05 was considered as statistical significant. IL, interleukin; TNF–α ,tumor necrosis factor alpha; INF–γ, interferon gamma; MMP–8, matrix metalloproteinase–8, TIMP–1, metallopeptidase inhibitor–1, ICAM–1, intracellular adhesion molecule–1; ANG–2:ANG1, angiopoetin–2: angiopoetin–1, PAI–1, Plasminogen activator inhibitor–1. NA, not applicable. | | |

**Table S18**. Mediation pathway analysis – age, systemic biomarkers and mortality, multiple regression models of patients ARDS without CCI and comorbidity as covariates *(Mediation step 4)*

|  | **Multiple regression models^‡^** | | | | | |  |  |
| --- | --- | --- | --- | --- | --- | --- | --- | --- |
|  | **Total effect [95% CI]** | **ADE [95% CI]** | **p–value** | **ACME [95% CI]** | **p–value** | **Proportion of mediation**  **[95% CI]** | **p-value** | **p-value, BH correction** |
| **Fractalkine** |  |  |  |  |  |  |  |  |
| **Middle–aged** | 0.09 [0.00, 0.18] | 0.10 [0.02, 0.19] | 0.028 | –0.01 [–0.03, 0.00] | 0.094 | –0.10 [–1.81, 0.54] | 0.258 | 0.258 |
| **Elderly** | 0.17 [0.10, 0.26] | 0.19 [0.10, 0.27] | <0.001 | –0.02 [–0.04, –0.00] | 0.020 | –0.11 [–0.57, –0.02] | 0.028 | 0.056 |
| **tPA** |  |  |  |  |  |  |  |  |
| **Middle–aged** | 0.09 [0.00, 0.18] | 0.09 [–0.01, 0.16] | 0.076 | 0.00 [–0.01, 0.02] | 0.562 | 0.04 [–0.37, 0.59] | 0.086 | 0.115 |
| **Elderly** | 0.16 [0.06, 0.25] | 0.14 [0.05, 0.23] | 0.002 | 0.02 [0.00, 0.03] | 0.024 | 0.13 [0.01, 0.38] | 0.028 | 0.056 |

Data is presented as estimate with a 95 % confidence interval [CI]. ADE, average direct effect; ACME, average causal mediation effect. Adjusted for: ethnic background, gender, admission type, readmission, direct–hit for ARDS, immunodeficiency, tidal volume per predicted body weight, positive end expiratory pressure. A p–value < 0.05 was considered as statistically significant. There was no significant exposure–mediator interaction for any mediator. BH, Benjamini-Hochberg , IL–8, Interleukin–8; IL–10, Interleukin–10; PF4, platelet factor 4; PAI–1, Plasminogen activator inhibitor–1; tPA, tissue plasminogen activator.

***Sensitivity analysis with age as a continuous variable***

**Table S19** Association between age (continuous variable) and mortality (*Mediation step 1*)

|  | **Crude odds ratio [95% CI]** | **p–value** | **Adjusted odds ratio**  **[95% CI]** **‡** | **p–value** | **GOF p-value** |
| --- | --- | --- | --- | --- | --- |
| **30 day mortality** | 1.01 [1.00, 1.03] | 0.04 | 1.02 [1.00, 1.03] | 0.01 | 0.04 |
| **90 day mortality** | 1.02 [1.00, 1.03] | <0.01 | 1.02 [1.01, 1.04] | <0.01 | 0.14 |
| **1 year mortality** | 1.02 [1.01, 1.04] | <0.01 | 1.03 [1.02, 1.04] | <0.01 | 0.25 |
| Data is presented as odds ratio’s (OR) with a 95 % confidence interval [CI]. ‡ Adjusted for: ethnic background, gender, admission type, readmission, direct–hit for ARDS, Charlson Comorbidity Index, APACHE IV score adjusted for age, immunodeficiency, tidal volume per predicted body weight, positive end expiratory pressure. A p–value < 0.05 was considered as statistically significant. GOF = goodness of fit, omnibus test, a p-values of < 0.05 is considered as a lack of fit. NA = not applicable | | | | | |

**Table S20** Association between age (continuous variable) and inflammatory marker levels in ARDS patients *(Mediation Step 3)*

|  | **Coefficient [95% CI]** | |  | **Adjusted Coefficient [95% CI]** |  | **Adjusted R^2^** |
| --- | --- | --- | --- | --- | --- | --- |
| **Inflammation** |  | | P–value |  | P–value | **p-value** |
| **IL–6** | –0.01 [–0.02, –0.00] | | 0.04 | –0.01 [–0.02, 0.00] | 0.06 | 0.16 |
| **IL–8** | –0.01 [–0.02, –0.00] | | 0.01 | –0.00 [–0.02, 0.00] | 0.16 | 0.25 |
| **IL–10** | –0.01 [–0.02, –0.00] | | <0.01 | –0.01 [–0.02, –0.00] | 0.04 | 0.20 |
| **IL1–β** | –0.00 [–0.00, 0.00] | | 0.91 | 0.00 [–0.00, 0.01] | 0.47 | 0.06 |
| **TNFα** | –0.00 [–0.00, 0.01] | | 0.31 | 0.00 [–0.00, 0.01] | 0.19 | 0.01 |
| **IFNγ** | –0.01 [–0.02, 0.00] | | 0.07 | –0.00 [–0.01, 0.00] | 0.23 | 0.03 |
| **MMP–8** | –0.00 [–0.01, 0.01] | | 0.57 | –0.00 [–0.02, 0.01] | 0.51 | 0.05 |
| **TIMP–1** | –0.00 [–0.01, 0.00] | | 0.48 | 0.00 [–0.01, 0.01] | 0.95 | 0.12 |
| **Endothelial activation markers** |  | |  |  |  |  |
| **Fractalkine** | –0.01 [–0.02, –0.01] | | <0.01 | –0.01 [–0.0, –0.00] | <0.01 | 0.19 |
| **E–selectin** | –0.01 [–0.02, 0.00] | | <0.01 | –0.01 [–0.01, 0.00] | 0.09 | 0.04 |
| **P–selectin** | 0.00 [–0.01, 0.01] | | 0.75 | 0.00 [–0.00, 0.01] | 0.59 | 0.06 |
| **ICAM–1** | | –0.00 [–0.01, 0.00] | 0.05 | –0.00 [–0.01, 0.00] | 0.45 | 0.08 |
| **ANG2:ANG1** | | 0.00 [–0.02, 0.01] | 0.47 | –0.00 [–0.02, 0.01] | 0.53 | 0.05 |
| **Coagulation** | |  |  |  |  |  |
| **PF4** | | 0.01 [–0.01, 0.02] | 0.27 | 0.00 [–0.01, 0.02] | 0.82 | 0.13 |
| **DDimer** | | 0.00 [–0.01, 0.01] | 0.96 | –0.00 [–0.00, 0.01] | 0.80 | 0.02 |
| **tPA** | | 0.00 [–0.00, 0.01] | 0.09 | 0.01 [0.00, 0.01] | 0.03 | 0.07 |
| **PAI­–1** | | –0.01 [–0.02, –0.00] | 0.02 | –0.01 [–0.02, 0.00] | 0.11 | 0.14 |
| **Protein C** | | –0.00 [–0.00, 0.00] | 0.06 | –0.00 [–0.01, –0.00] | 0.05 | 0.03 |
| **AT** | | –0.00 [–0.01, 0.00] | 0.10 | –0.00 [–0.01, 0.00] | 0.17 | 0.06 |

Data is presented as beta–coefficient (β) with a 95 % confidence interval [CI]. All biomarkers are log–transformed. **^*^‡** Adjusted for: ethnic background, gender, admission type, readmission, Charlson Comorbidity Index, APACHE–IV score adjusted for age, immunodeficiency, tidal volume per predicted body weight, positive end expiratory pressure. A p–value < 0.05 was considered as statistical significant. IL, interleukin; TNF–α ,tumor necrosis factor alpha; INF–γ, interferon gamma; MMP–8, matrix metalloproteinase–8, TIMP–1, metallopeptidase inhibitor–1, ICAM–1, intracellular adhesion molecule–1; ANG–2:ANG1, angiopoetin–2: angiopoetin–1, PAI–1, Plasminogen activator inhibitor–1. Goodness of fit, adjusted R2, a p-values of < 0.05 is considered as a lack of fit.

|  | **Total effect [95% CI]** | **ADE [95% CI]** | **p–value** | **ACME [95% CI]** | **p–value** | **Proportion of mediation [95% CI]** | **p-value** | **p-value, BH correction** |
| --- | --- | --- | --- | --- | --- | --- | --- | --- |
| **IL–6** |  |  |  |  |  |  |  |  |
| **Univariate** | 0.0022 [0.0010, 0.00028] | 0.0025 [0.0013, 0.0031] | 0.004 | –0.0002 [–0.0006, 0.0000] | 0.048 | –0.09 [–0.51, 0.00] | 0.058 | 0.070 |
| **Adjusted** | 0.0025 [0.0017, 0.0029] | 0.0026 [0.0018, 0.0031] | <0.001 | –0.0000 [–0.0003, 0.0000] | 0.126 | –0.03 [–0.13, 0.01] | 0.128 | 0.154 |
| **IL–8** |  |  |  |  |  |  |  |  |
| **Univariate** | 0.0024 [0.0013, 0.0029] | 0.0029 [0.0019, 0.0036] | <0.001 | –0.0005 [–0.0011, –0.0000] | 0.010 | –0.20 [–0.1.00, –0.02] | 0.016 | 0.048 |
| **Adjusted** | 0.0025 [0.0017, 0.0029] | 0.0027 [0.0019, 0.0032] | <0.001 | –0.0002 [–0.0005, 0.0000] | 0.172 | –0.05 [–0.24, 0.02] | 0.176 | 0.176 |
| **IL–10** |  |  |  |  |  |  |  |  |
| **Univariate** | 0.0023 [0.0012, 0.0029] | 0.0028 [0.0019, 0.0035] | 0.002 | –0.0005 [–0.0010, –0.0001] | 0.016 | –0.17 [–0.88, –0.02] | 0.028 | 0.051 |
| **Adjusted** | 0.0025 [0.0017, 0.0029] | 0.0027 [0.0019, 0.0032] | 0.002 | –0.0002 [–0.0005, 0.0000] | 0.059 | –0.07 [–0.25, 0.00] | 0.068 | 0.136 |
| **Fractalkine** |  |  |  |  |  |  |  |  |
| **Univariate** | 0.0024 [0.0013, 0.0029] | 0.0033 [0.0025, 0.0040] | <0.001 | –0.0009 [–0.0017, –0.0003] | <0.001 | –0.36 [–1.42, –0.13] | 0.004 | 0.024 |
| **Adjusted** | 0.0025 [0.0017, 0.0029] | 0.0029 [0.0022, 0.0034] | <0.001 | –0.0003 [–0.0008, –0.0000] | 0.013 | –0.12 [–0.40, –0.03] | 0.012 | 0.060 |
| **tPA** |  |  |  |  |  |  |  |  |
| **Univariate** | 0.0023 [0.0011, 0.0029] | 0.0020 [0.0008, 0.0027] | 0.012 | 0.0003 [–0.0001, 0.0007] | 0.146 | 0.12 [-0.05, 0.40] | 0.152 | 0.152 |
| **Adjusted** | 0.0025 [0.0017, 0.0029] | 0.0023 [0.0014, 0.0028] | 0.002 | 0.0002 [0.0000, 0.0006] | 0.028 | 0.09 [0.01, 0.25] | 0.020 | 0.060 |
| **PAI–1** |  |  |  |  |  |  |  |  |
| **Univariate** | 0.0024 [0.0011, 0.0029] | 0.0028 [0.0019, 0.0036] | <0.001 | –0.0005 [–0.0011, –0.0000] | 0.037 | –0.19 [–0.80, –0.01] | 0.034 | 0.051 |
| **Adjusted** | 0.0025 [0.0019, 0.0030] | 0.0028 [0.0021, 0.0034] | <0.001 | –0.0002 [–0.0006, 0.0000] | 0.114 | –0.07 [–0.30, 0.01] | 0.116 | 0.153 |

**Table S21** Mediation pathway analysis – age (continuous variable), systemic biomarkers and mortality, univariate and multiple regression models *(Mediation step 4)*

Data is presented as estimate with a 95 % confidence interval [CI]. ADE, average direct effect; ACME, average causal mediation effect. Adjusted for: ethnic background, gender, admission type, readmission, Charlson Comorbidity Index, APACHE IV score adjusted for age, direct-hit for ARDS immunodeficiency, tidal volume per predicted body weight, positive end expiratory pressure. A p–value < 0.05 was considered as statistical significant. There was no significant exposure–mediator interaction for any mediator. BH, Benjamini-Hochberg; IL–6, Interleukin–6; IL–8, Interleukin–8; IL–10, Interleukin–10; PAI-1, Plasminogen activator inhibitor–1; tPA, tissue plasminogen activator.

***Subgroup analysis: Exploration of age-related temporal differences***

**Table S22** Covariates of patients with a sample at a later time point.

|  | **Young adults**  **(n=119)** | | **Middle–age adults**  **(n=1110**) | | **Elderly**  **(n=121**) | | **p–value** |
| --- | --- | --- | --- | --- | --- | --- | --- |
| Time to sample from the onset of ARDS, Median [IQR] | 5 | [4, 7] | 4 | [4, 6] | 4 | [4, 7] | 0.26 |
| Age (years), Median [IQR] | 44 | [35, 50] | 62 | [59, 64] | 73 | [71, 77] | <0.01 |
| Male, n/total n (%) | 63 | (53) | 36 | (33) | 80 | (66) | 0.04 |
| Race, Caucasian, n/total n (%) | 93  93 | (87) | 98 | (89) | 116 | (96) | <0.01 |
| Admission type, Medical, n/total n (%) | 95 | (80) | 84 | (76) | 84 | (69) | 0.16 |
| Readmission, n/total n (%) | 13 | (11) | 18 | (16) | 21 | (17) | 0.35 |
| Charlson Comorbidity Index, Median [IQR] | 1 | [0, 2] | 0 | [0, 2] | 0 | [0, 2] | 0.46 |
| APACHE IV Score adjusted for age, Median [IQR] | 77 | [58, 101] | 75 | [56, 94] | 70 | [51, 85] | 0.08 |
| Tidal volume per PBW, Median [IQR] | 7.2 | [6.1, 8.1] | 7.0 | [6.2, 8.1] | 7.1 | [6.3, 8.0] | 0.87 |
| PEEP (cmH_2_O), Median [IQR] | 10 | [8, 14] | 10 | [8, 12] | 10 | [7, 12] | 0.09 |
| PaO_2_ FiO_2_ ratio, Median [IQR] | 167 | [122, 228] | 160 | [111, 206] | 157 | [118, 216] | 0.48 |

IQR, interquartile range; APACHE, acute physiology and chronic health evaluation; PBW, predicted body weight; PEEP, positive end-expiratory pressure; A p–value < 0.05 was considered as statistically significant.

**Figure S3.** Biomarker levels at a later timepoint, stratified by age-groups


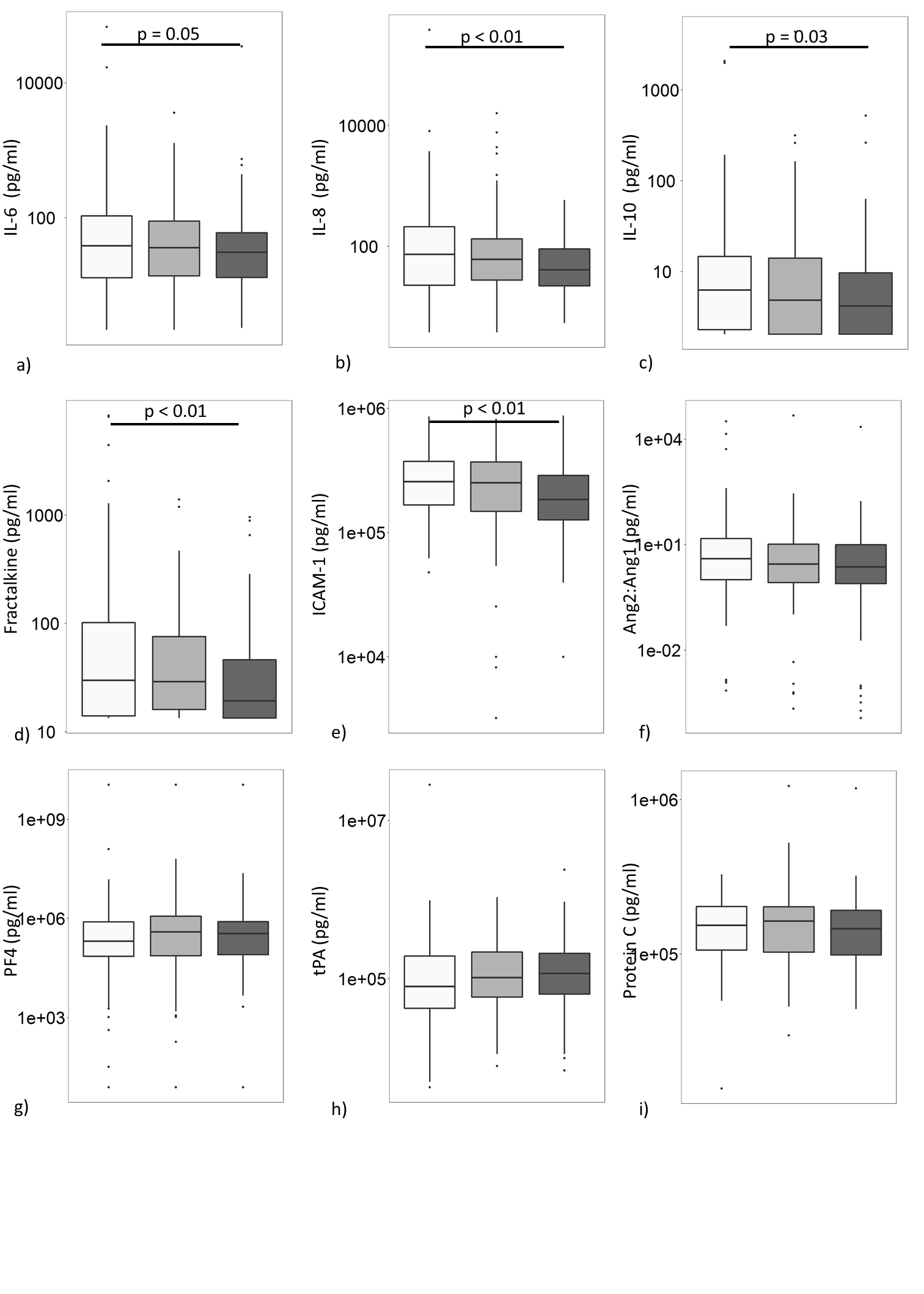


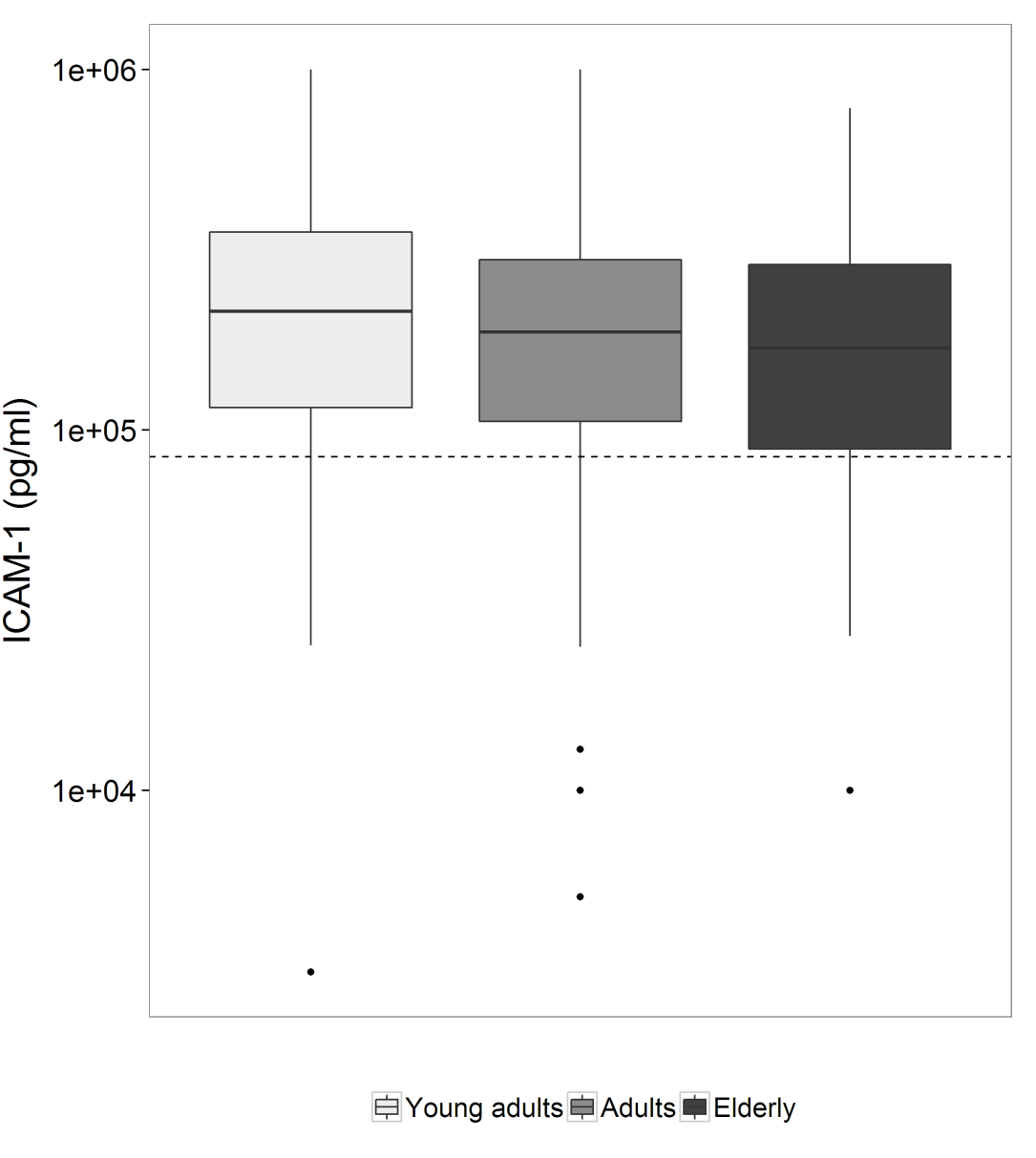
**
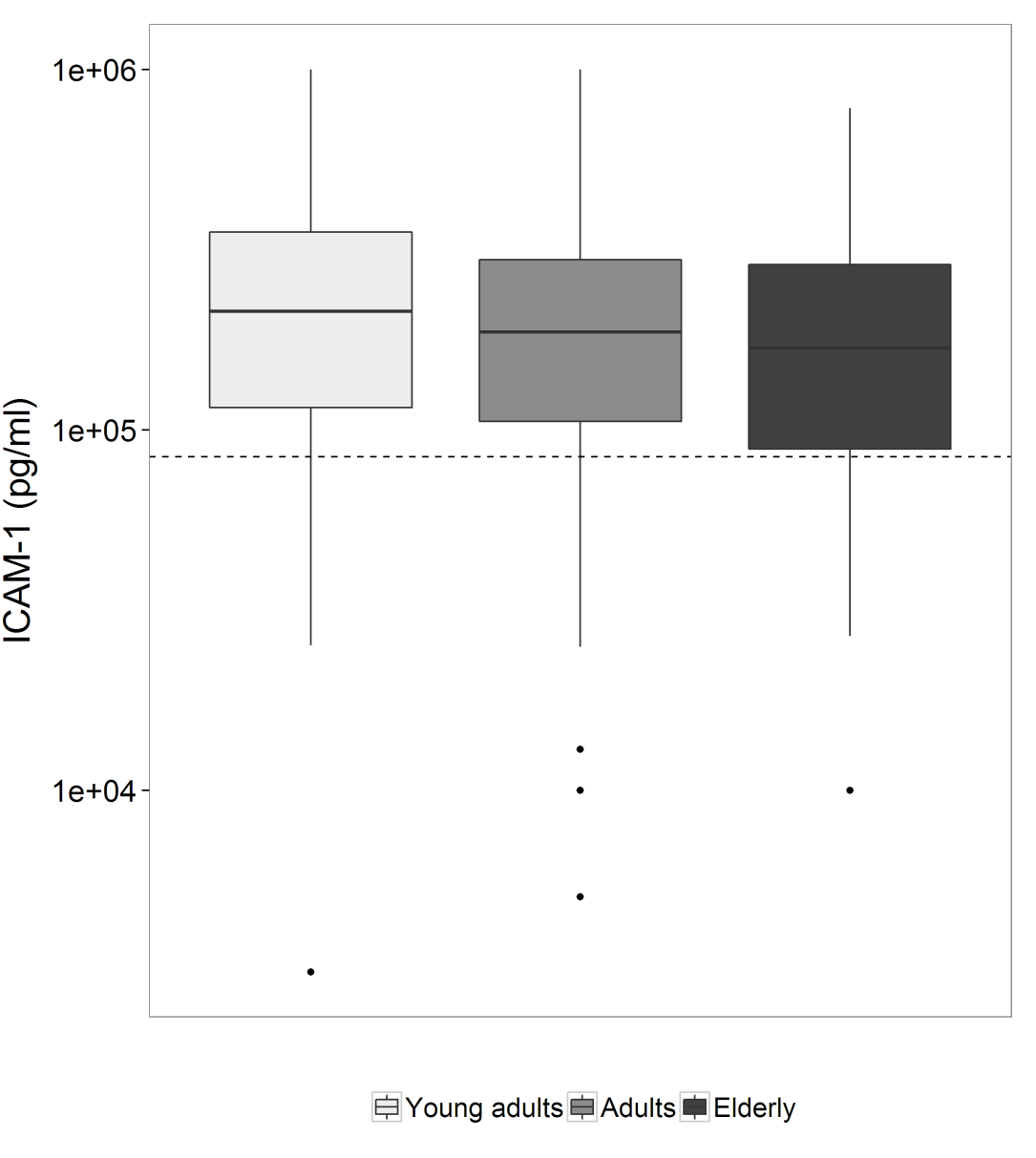

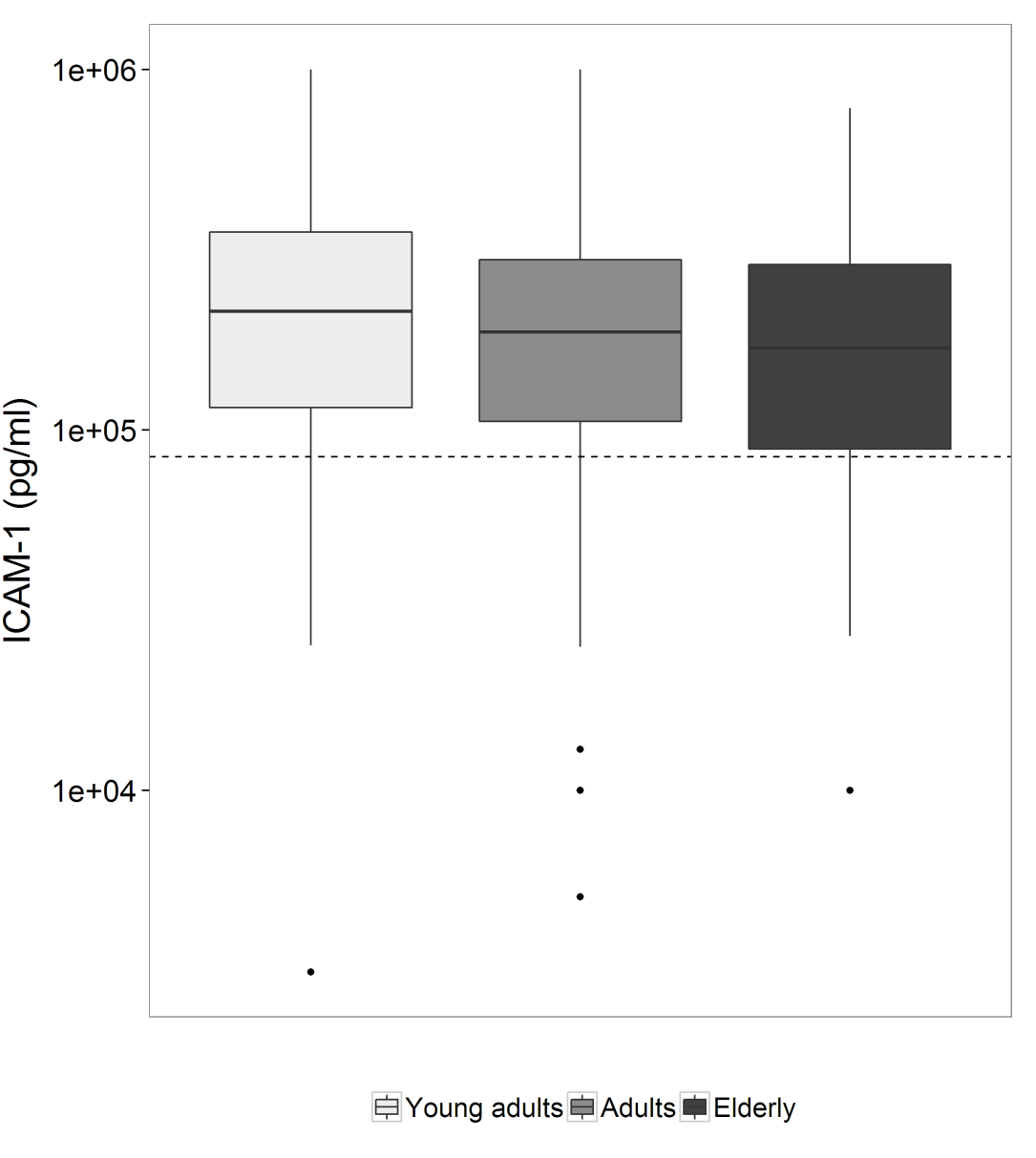
**Systemic levels of a) Interleukin (IL)–6, b) IL–8, c) IL–10, d) Fractalkine, e) Intracellular adhesion molecule (ICAM)–1, f) Angiopoetin 2: Angiopoetin 1 (ANG2:ANG1), g) Platelet factor (PF) 4, h) Tissue plasminogen activator (tPA), i) protein–C, at onset of ARDS. Young adults, Middle aged adults , Elderly. Box and whisker diagrams depict the median and lower quartile, upper quartile, and their respective 1.5 IQR as whiskers – as specified by Tukey. Group differences between young adults and middle aged adults, and young adults and elderly were tested by a Mann–Whitney U test. A p–value < 0.05 was considered as statistical significant.

**
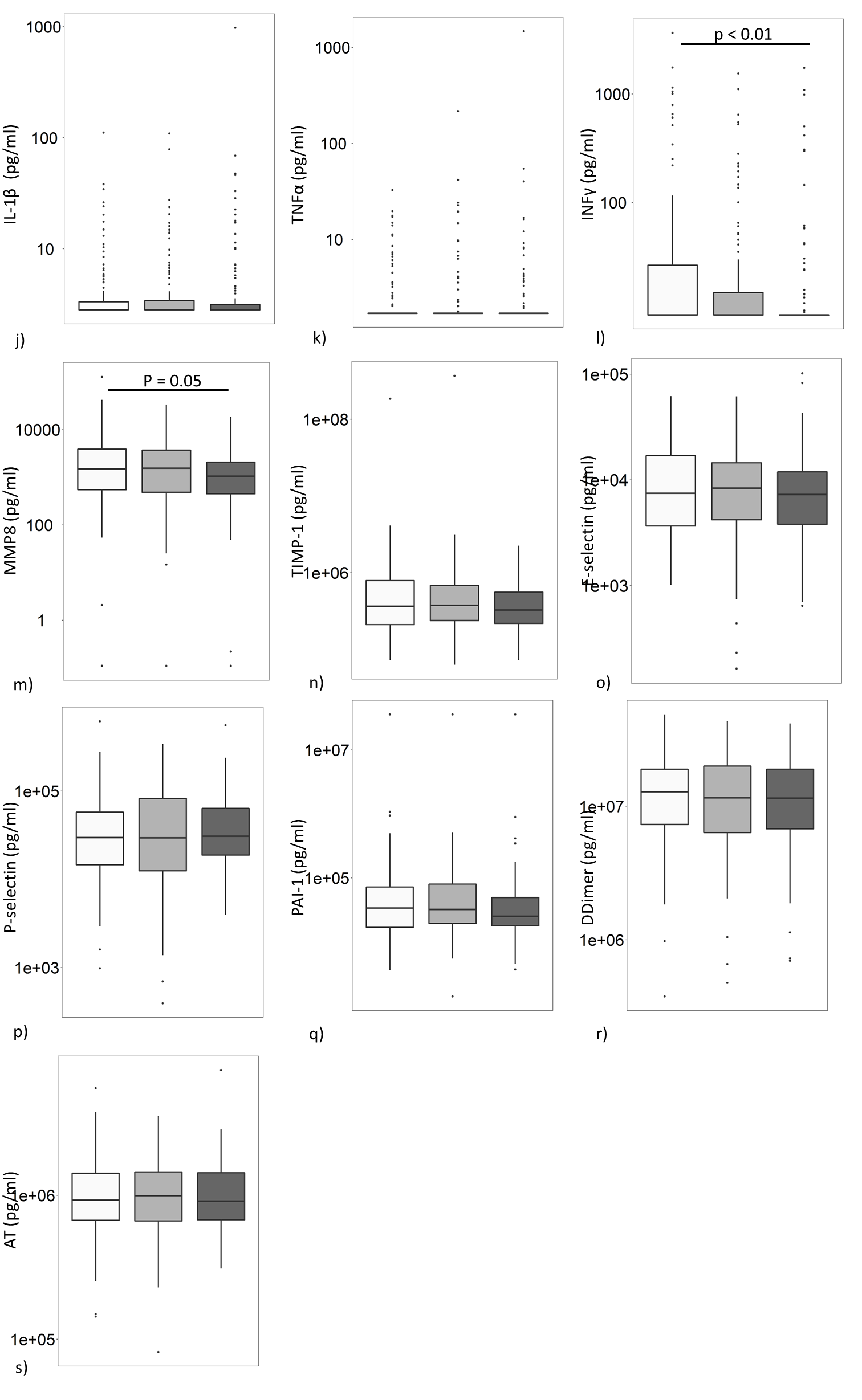
**

**
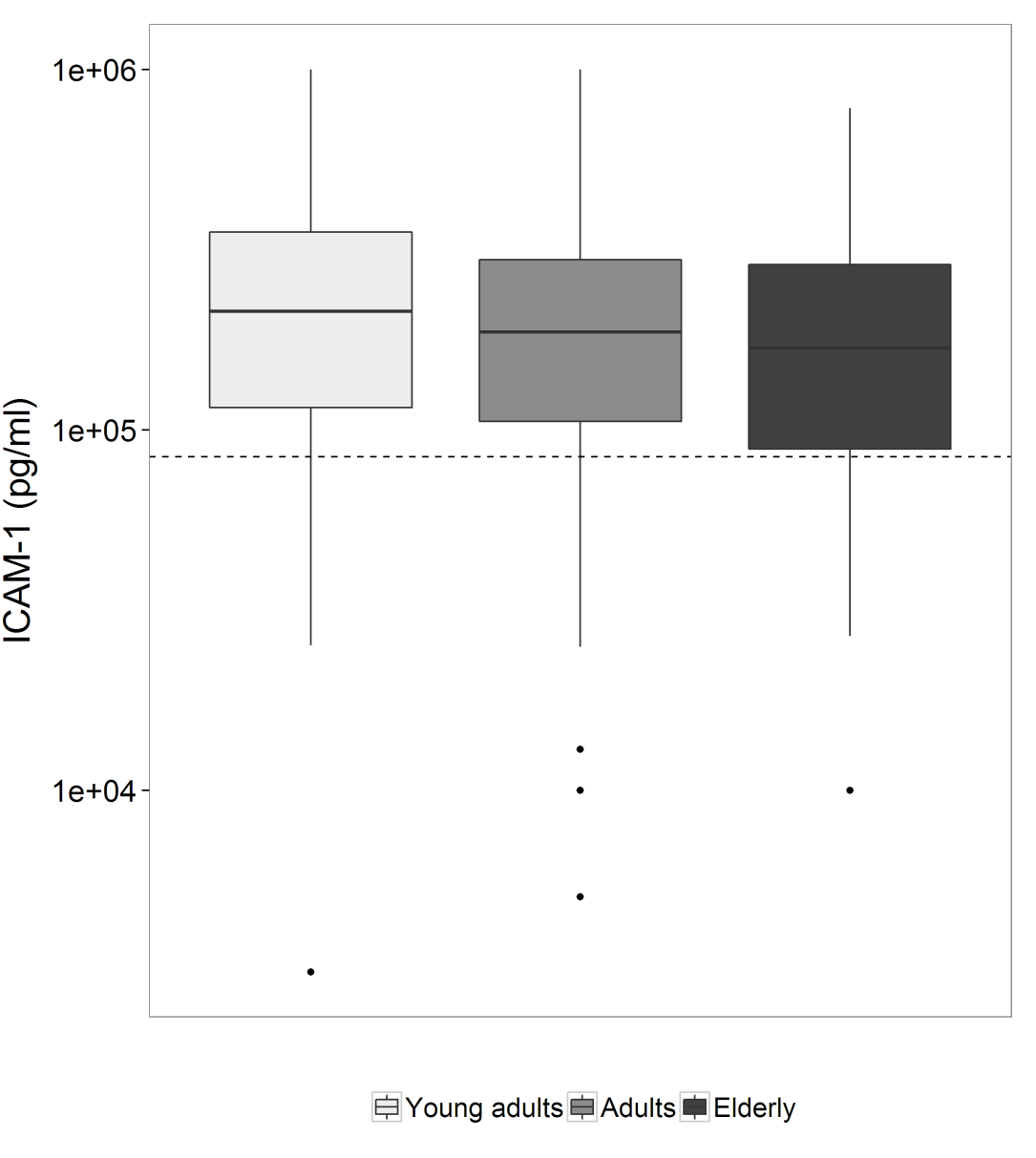

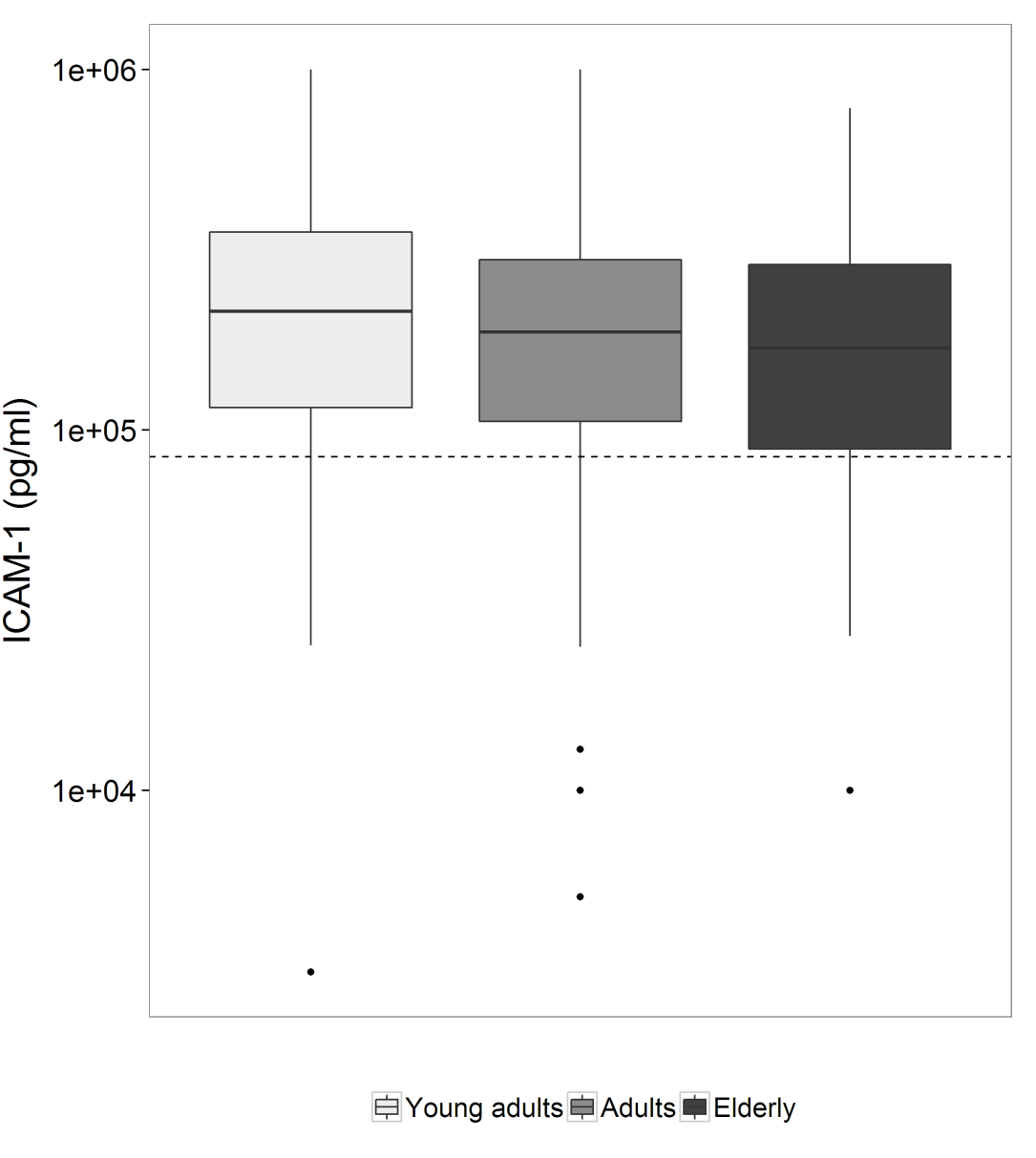

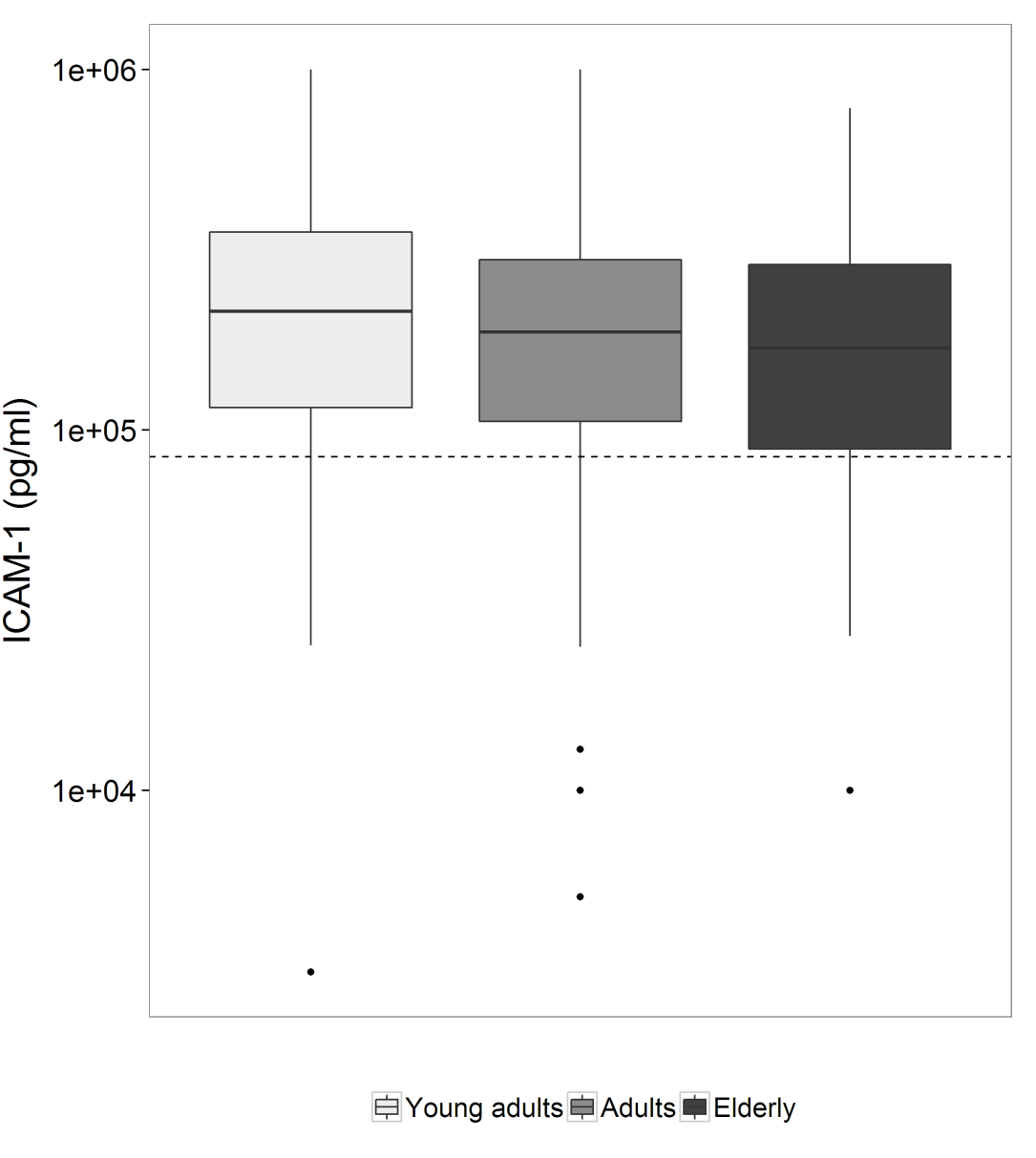
**Systemic levels of j) Interleukin (IL)–1β k) tumor necrosis factor (TNF)–α l) Interferon (INF)–γ, m) matrix metalloproteinase (MMP)–8, n) metallopeptidase inhibitor (TIMP)–1, o) E–selectin, p) P–selectin, q) Plasminogen activator inhibitor (PAI)–1, r) DDimer, s) Anti–thrombin (AT) . Young adults Middle aged adults Elderly. Box and whisker diagrams depict the median and lower quartile, upper quartile, and their respective 1.5 IQR as whiskers – as specified by Tukey. Group differences between young adults and middle aged adults, and young adults and elderly were tested by a Mann–Whitney U test. A p–value < 0.05 was considered as statistically significant.

**Table S23** Results of the literature review.

| Author (year of publication) | Patient population | Measures | Outcome |
| --- | --- | --- | --- |
| Stanojcic et al (2016) | Burn injury | Inflammatory mediators in serum | Elderly burned patients mount a delayed immune and dampened inflammatory response early after burn injury that changes to an augmented response at later time points. |
| Jeschke et al (2015) | Burn injury | Inflammatory mediators in serum and monocyte HLA–DR. | Elderly burned patients had an early hypo–inflammation followed by a hyper–inflammation after 2 weeks post–burn. |
| Vieira da Silva Pellegrina et al (2015) | Sepsis | Transcriptomics: gene expression profiles of neutrophils | Major pathways, included in the pathways of oxidative phosphorylation, mitochondrial dysfunction and TGF–β signalling, among others, were preferentially deregulated in the elderly compared to young adults. |
| Ginde et al (2014) | Sepsis | Inflammatory mediators in serum | Older adults had higher levels of inflammation at presentation, these age–related differences in inflammation largely resolved during the first 72 h of hospitalization. |
| Inoue et al (2014) | Severe sepsis | Neutrophils, T cells and inflammatory mediators in serum. | No difference in neutrophil counts, but persistent increased levels of IL–6 and T cell exhaustion in elderly compared to young adults |
| Cao et al (2014) | Severe sepsis | Semi–quantitative plasma proteomics | Proteins involved in the pathways (acute phase response, coagulation, lipid metabolism, atherosclerosis, and production of NO and ROS) show opposite expression levels dependent on patient age. |
| Marik et al (2001) | Septic shock | Inflammatory mediators in serum | IL–6, sTNF–R55, and sTNF–R75 levels were similar among the different age groups. The TNF–alpha levels were significantly higher, however, in the oldest group of patients. |
| Menter et al (2014) | Streptococcus pneumonia | Lymphocytes, neutrophils and macrophages in lung tissue | Higher percentage of neutrophilic granulocytes, less alveolar macrophages in elderly compared to young patients. |
| Kale et al (2010) | Community–acquired pneumonia | Inflammatory mediators, cell–surface markers and coagulation markers in serum | No age–related differences in inflammatory and cell surface markers occurred during the first week after admission. But higher levels of pro–coagulant markers in older patients compared to young. |
| Kelly et al (2009) | Community–acquired pneumonia | Inflammatory mediators in serum | IL–6 and IL–10 levels were similar in young (< 65 years), older (> 65 years) and very elderly (> 80 years) patients. |
| Glynn et al (1999) | Community–acquired pneumonia | Endothelial activation markers in plasma and CD11b positive neutrophils | No difference in sICAM–1, sE–selectin or CD11b positive neutrophils between adults and elderly. |
| Gon et al (1996) | Pneumonia | Inflammatory mediators in serum, and whole blood stimulations. | Lower levels of G–CSF, GM–CSF, IL–1β, TNF–α, IL–8 and MIP–1α and impaired production of these cytokines in the elderly compared to adults. |
| Bruunsgaard et al (1999) | Streptococcus pneumonia | Inflammatory mediators in serum | Levels of cytokines were similar on admission, but levels of TNF–α and sTNFR–I were higher after 1 week in elderly (68–91 years) compared to young, and the TNFa/ IL–10 ratio from day 0 to day 7 were correlated with age. |
| Boldt et al (1997) | Critically ill (APACHE II score > 15 points) | Endothelial activation markers in plasma | Higher levels of ICAM–1 and VCAM–1 and GMP–140 in elderly compared to adults. |

TGF–β, transforming growth factor beta; IL, Interleukin; NO, nitrogen oxide; ROS, reactive oxygen species; TNF, tumor necrosis factor; sICAM–1, soluble cell adhesion molecule–1; G–CSF, granulocyte colony–stimulating factor; GM–CSF, granulocyte macrophage colony–stimulating factor; MIP–1α, Macrophage Inflammatory Protein – 1alfa; VCAM–1, vascular cell adhesion molecule–1; GMP–140, platelet alpha–granule membrane protein–140; APACHE II score, Acute Physiology and Chronic Health Evaluation II score

**Reference**

1. Zimmerman JE, Kramer AA, McNair DS, Malila FM (2006) Acute Physiology and Chronic Health Evaluation (APACHE) IV: hospital mortality assessment for today’s critically ill patients. Crit Care Med 34:1297–310. doi:10.1097/01.CCM.0000215112.84523.F0

2. Gajic O, Dabbagh O, Park PK, et al (2011) Early identification of patients at risk of acute lung injury: evaluation of lung injury prediction score in a multicenter cohort study. Am J Respir Crit Care Med 183:462–70. doi:10.1164/rccm.201004-0549OC

3. Quan H, Li B, Couris CM, et al (2011) Updating and validating the Charlson comorbidity index and score for risk adjustment in hospital discharge abstracts using data from 6 countries. Am J Epidemiol 173:676–82. doi:10.1093/aje/kwq433

4. van Vught LA, Wiewel MA, Hoogendijk AJ, et al (2017) The Host Response in Patients with Sepsis Developing Intensive Care Unit-acquired Secondary Infections. Am J Respir Crit Care Med 196:458–470. doi:10.1164/rccm.201606-1225OC

5. IMAI K, KEELE L, TINGLEY D, YAMAMOTO T (2011) Unpacking the Black Box of Causality: Learning about Causal Mechanisms from Experimental and Observational Studies. Am Polit Sci Rev 105:765–789. doi:10.1017/S0003055411000414

6. Imai K, Keele L, Tingley D (2010) A general approach to causal mediation analysis. Psychol Methods 15:309–34. doi:10.1037/a0020761

7. Imai K, Keele L, Tingley D YT (2010) Causal mediation analysis using. In: Vinod HD (ed) Advances in social science research using R. pp 129–54

8. Tingley D, Yamamoto T, Hirose K, et al (2014) mediation : R Package for Causal Mediation Analysis. J Stat Softw 59:1–38. doi:10.18637/jss.v059.i05

9. Neto AS, Hemmes SNT, Barbas CS V, et al (2016) Association between driving pressure and development of postoperative pulmonary complications in patients undergoing mechanical ventilation for general anaesthesia: a meta-analysis of individual patient data. Lancet Respir Med 4:272–280. doi:10.1016/S2213-2600(16)00057-6

10. Valeri L, Vanderweele TJ (2013) Mediation analysis allowing for exposure-mediator interactions and causal interpretation: theoretical assumptions and implementation with SAS and SPSS macros. Psychol Methods 18:137–50. doi:10.1037/a0031034

11. Beulens JWJ, van der Schouw YT, Moons KGM, et al (2013) Estimating the mediating effect of different biomarkers on the relation of alcohol consumption with the risk of type 2 diabetes. Ann Epidemiol 23:193–7. doi:10.1016/j.annepidem.2012.12.014

12. Song Y, Huang YT, Song Y, et al (2015) Birthweight, mediating biomarkers and the development of type 2 diabetes later in life: a prospective study of multi-ethnic women. Diabetologia 58:1220–1230. doi:10.1007/s00125-014-3479-2
